# Fitting and Cross-Validating Cox Models to Censored Big Data With Missing Values Using Extensions of Partial Least Squares Regression Models

**DOI:** 10.3389/fdata.2021.684794

**Published:** 2021-11-01

**Authors:** Frédéric Bertrand, Myriam Maumy-Bertrand

**Affiliations:** ^1^ LIST3N, Université de Technologie de Troyes, Troyes, France; ^2^ IRMA, CNRS UMR 7501, Labex IRMIA, Université de Strasbourg, Strasbourg, France

**Keywords:** big data and analytics, censored data, partial least squares, sparse partial least squares regression, sparse partial least squares discriminant analysis, kernel techniques, cross validation, Cox models

## Abstract

Fitting Cox models in a big data context -on a massive scale in terms of volume, intensity, and complexity exceeding the capacity of usual analytic tools-is often challenging. If some data are missing, it is even more difficult. We proposed algorithms that were able to fit Cox models in high dimensional settings using extensions of partial least squares regression to the Cox models. Some of them were able to cope with missing data. We were recently able to extend our most recent algorithms to big data, thus allowing to fit Cox model for big data with missing values. When cross-validating standard or extended Cox models, the commonly used criterion is the cross-validated partial loglikelihood using a naive or a van Houwelingen scheme —to make efficient use of the death times of the left out data in relation to the death times of all the data. Quite astonishingly, we will show, using a strong simulation study involving three different data simulation algorithms, that these two cross-validation methods fail with the extensions, either straightforward or more involved ones, of partial least squares regression to the Cox model. This is quite an interesting result for at least two reasons. Firstly, several nice features of PLS based models, including regularization, interpretability of the components, missing data support, data visualization thanks to biplots of individuals and variables —and even parsimony or group parsimony for Sparse partial least squares or sparse group SPLS based models, account for a common use of these extensions by statisticians who usually select their hyperparameters using cross-validation. Secondly, they are almost always featured in benchmarking studies to assess the performance of a new estimation technique used in a high dimensional or big data context and often show poor statistical properties. We carried out a vast simulation study to evaluate more than a dozen of potential cross-validation criteria, either AUC or prediction error based. Several of them lead to the selection of a reasonable number of components. Using these newly found cross-validation criteria to fit extensions of partial least squares regression to the Cox model, we performed a benchmark reanalysis that showed enhanced performances of these techniques. In addition, we proposed sparse group extensions of our algorithms and defined a new robust measure based on the Schmid score and the R coefficient of determination for least absolute deviation: the integrated R Schmid Score weighted. The R-package used in this article is available on the CRAN, http://cran.r-project.org/web/packages/plsRcox/index.html. The R package bigPLS will soon be available on the CRAN and, until then, is available on Github https://github.com/fbertran/bigPLS.

## 1 Introduction

Standard PLS regression is an efficient tool to find the fundamental relations between two matrices (*X* and *Y*) by applying a latent variable approach to modelling the covariance structures in these two spaces. A PLS regression model will try to find the multidimensional direction iteratively in the *X* space that explains the maximum multidimensional variance direction in the *Y* space. A critical step in PLSR is to select the correct unknown number of these latent variables (called components) to use. If the predictors’ matrix has more variables than observations or feature multicollinearity among the *X* matrix columns, then standard —non regularized—regression will fail. On the contrary, PLS regression can cope with those settings.

PLS has become an established tool in various experimental settings such as chemometric, networks, or systems biology. This modelling is primaliry used because it is often possible to explain the underlying system’s extracted components and hence translate “hard” modelling information from the soft model: chemical components for NIR spectra, gene subnetwork for GRN or biological function for systems biology. As a consequence, choosing the right number of components is not only a major aim to avoid under or overfitting and ensure a relevant modeling or good predicting ability but also *per se*.

Relating personalized information from subjects such as omics data and subject survival or time to cancer recurrence is the focus of a vast literature from the last decade. The discovery of markers from big data or high-dimensional data, such as transcriptomic or SNP profiles, is a significant challenge in searching for more precise diagnoses. The most commonly used model for the analysis of survival data is the proportional hazard regression model suggested by Cox, 1972. Such a model helps the practitioner study in the presence of censoring the relationship between the time to event and a set of covariates. It has similar requirements as multivariate regression: more observations than variables, complete data, and not strongly correlated variables. In practice, when dealing with high-dimensional data, these constraints are crippling.

Missing Completely At Random (MCAR), Missing At Random (MAR), and Missing Not At Random (MNAR) are the three categories of missing data that were defined by Little and Rubin (2002). The data will be MCAR if the probability that the data is known depends neither on the observed value nor on the missing values. In the case of MAR, missingness depends only on the values of the observed data. Lastly, if missingness depends on the observed and missing data values, data are said to be MNAR.

Missing data imputation is a burning issue in statistics for any data size: from small to big data. For several years, many methods have been proposed to deal with missing values. There are various imputation methods from single value imputation, e.g., the mean over the complete cases in the study sample—known as mean imputation (Troyanskaya et al., 2001) to more complex methods, that include imputation based on Non-linear Iterative PArtial Least Squares (NIPALS) (Tenenhaus, 1998; Nengsih et al., 2019).

In this article, we deal with several PLS regression-based extensions of the Cox model that were first introduced in (Bastien, 2008) and (Bastien et al., 2015) and extend them twice: to group and sparse group models and to big data. These extensions share features praised by practitioners, including regularization, interpretability of the components, missing data support, biplots of individuals and variables —and even parsimony for SPLS based models—, and allow to deal with highly correlated predictors or even rectangular datasets, which is especially relevant for high dimensional datasets.

## 2 Models

### 2.1 Modeling Censored Data

#### 2.1.1 The Cox Proportional Hazards Model

Let assume the hazard function for the occurrence of an event —for instance, death or cancer relapse-at time *t* in the presence of censoring:

$$\lambda \left(t\right)=\lambda_{0}\left(t\right)\exp\left(\beta^{\prime }X\right),$$ (1)

where *λ*_0_(*t*) is an unspecified baseline hazard function, *β* is the vector of the coefficients and *X* the model matrix. Based on the available data, Cox’s partial likelihood can be written as:

$$PL\left(\beta \right)=\underset{k\in D}{\prod }\frac{\exp\left(\beta^{\prime }x_{k}\right)}{\sum_{j\in R_{k}}\exp\left(\beta^{\prime }x_{j}\right)},$$ (2)

where *D* is the set of indices of the events and *R*_*k*_ denotes the set of indices of the individuals at risk at time *t*_*k*_. This log partial likelihood function is not uniquely maximized if *p* > *n*. There may still be issues if *p* ⩽ *n* since covariates could be highly correlated. As a consequence, regularization may still be required in order to improve the predictive performance and to reduce the variances of the estimates.

#### 2.1.2 Deviance Residuals

As for many other statistical models, the Cox models’ residuals are of particular relevance yet more complicated than those coming from linear models. There are several kinds of such residuals: for instance, martingale residuals or deviance residuals. In this article, we will extend an idea from our previous work Bastien et al. (2015) where we used deviance residuals as a mean to apply PLS or SPLS to censored data.

Let define the event status *δ*_*i*_ for the *i*th subject with observation time *t*_*i*_ by *δ*_*i*_ = 0 if *t*_*i*_ is a censored time, and *δ*_*i*_ = 1 otherwise is. The martingale residuals for the *i*th subject for the Cox model with no time-dependent explanatory variables and at most one event per patient is:

$${\hat{M}}_{i}=\delta_{i}-{\hat{E}}_{i}=\delta_{i}-{\hat{\Delta }}_{0}\left(t_{i}\right)\exp\left(\hat{\beta }\prime x_{i}\right)$$ (3)

with ${\hat{\Delta }}_{0}\left(t_{i}\right)$ the estimated cumulative hazard function at time *t*_*i*_.

It is a common property that martingale residuals are highly skewed. As a consequence, a normalized transform of those residuals was defined and called deviance residuals. For the Cox model, the deviance residuals (Collett, 1994) *d*_*i*_ is:

$$d_{i}=sign\left({\hat{M}}_{i}\right)\cdot {\left[2\left\{-{\hat{M}}_{i}-\delta_{i}\log\left(\frac{\delta_{i}-{\hat{M}}_{i}}{\delta_{i}}\right)\right\}\right]}^{1/2}\cdot$$ (4)

More details on how to decipher that transform can be found in Bastien et al. (2015). In a word, the deviance residual, as a measure of excess of death, can be interpreted as a hazard measure.

### 2.2 PLS Regression Models and Extensions

#### 2.2.1 PLSR

PLS regression can be viewed as a regularization method based on dimension reduction. It was developed as a chemometric tool to find reliable predictive models with spectral data (Wold et al., 1983; Tenenhaus, 1998). Nowadays, using huge matrices for classification or prediction still raise similar issues. As a result, PLS regression principles were put in use in this new context. It aims to find linear combinations of the original variables —latent variables— and use them as new descriptors in standard regression analysis. This method uses the response variable in constructing the latent components, unlike principal components analysis (PCA). It can be viewed as a regularized approach giving biased regression coefficients but with lower variance. The NIPALS algorithm allows fitting PLS regression models on datasets with missing data.

#### 2.2.2 Sparse (Group) PLSR

A large number of predictors affect PLS regression’s performance (Chun and Keles, 2010). Besides, in the linear regression setting, coefficient estimates’ inconsistency often occurs due to a high number of irrelevant variables. As a consequence, filtering is a usually required preprocessing step before PLS fit. Before Chun and Keles proposed “sparse PLS regression”, commonly used filtering approaches were all univariate. sPLS promotes variables selection as the PLS dimension reduction is being applied and can include variables that variable filtering would select in constructing the first direction vector. Imposing *L*_1_ constraint on PLS direction vector *w* defines a direct extension of PLS regression to sPLS regression:

$$\begin{array}{ll} & \underset{w}{\max}w\prime Mw\;\text{subject to }w\prime w=\|w{\|}_{2}=1,\|w{\|}_{1}⩽\lambda ,\\ & \text{where }M=X\prime YY\prime X. \end{array}$$

However, for *Y* = *X*, it is known that the problem is equivalent to sPCA (Jolliffe et al., 2003), which is not convex and that the solution is often not sparse enough. Chun and Keles used the LARS algorithm to solve these issues by extending the regression formulation of sPCA of Zou et al., 2006:

$$\begin{array}{ll} & \underset{w,c}{\min}-\kappa w\prime Mw+\left(1-\kappa \right)\left(c-w\right)\prime M\left(c-w\right)+\lambda_{1}\|c{\|}_{1}+\lambda_{2}\|c{\|}_{2}\\ & \text{subject to }w\prime w=1,\text{where }M=X\prime YY\prime X. \end{array}$$

The use of a surrogate *w* of the direction vector *c* and both *L*_1_ and *L*_2_ penalties favour exact zero property and take care of the potential singularity of *M*. For univariate PLS, *y* regressed on *X*, Chun and Keles derived the first direction vector by soft thresholding of the original PLS direction vector:

$${\left(|Z|-\frac{\lambda }{2}\right)}_{+}sign\left(Z\right),\text{ where }Z=X\prime y/\|X\prime y{\|}_{2}.$$ (5)

sPLS achieves fast convergence by using conjugate gradient. The computational cost for computing coefficients at each step of the sPLS algorithm is less than or equal to the computational cost of computing step size in LARS since conjugate gradient methods avoid matrix inversion.

PLS regression, sparse PLS regression and sparse group PLS regression were recently extended to big data in a scalable way (de Micheaux et al., 2019). We adapted the algorithms used by these authors to cope with missing values.

### 2.3 Extensions of PLSR Models to Censored Data

#### 2.3.1 PLS-Cox

There are several algorithms to fit PLS regression models. A succession of simple and multiple linear regressions may be employed (Garthwaite, 1994). A NIPALS-like (Wold, 1966) algorithm was derived by Tenenhaus (1999) to fit PLS regression models coping with missing data. Bastien and Tenenhaus (2001) used a similar idea to extend PLS regression to any generalized linear regression model (PLS-GLR), the Cox model (PLS-Cox) being a particular case. Using this equivalence between Cox models and some GLR models, we were able to adapt these algorithms and further developments from (Bastien et al., 2005) to fit big data (Gentleman (1982), Miller (1992), Gentleman, 1974, Gentleman (1982), Miller (1992); Miller, 1994). More details on these extensions of PLS regression to Cox models and their comparison to other extensions can be found in (Bastien et al., 2015).

#### 2.3.2 (DK) (S) (G)PLS(DR)

##### 2.3.2.1 The PLSDR Algorithm

PLSDR is an alternative in high-dimensional settings using deviance residuals based PLS regression, advantageous both by its simplicity and efficiency (Bastien, 2008): first compute null deviance residuals using a simple Cox model without covariates, then fit a standard PLS regression using them as an outcome. The *m* retained PLSDR components are used to fit the final Cox model.

This algorithm was implemented in the plsRcox R package (Bertrand et al., 2014; Bastien et al., 2015; Bertrand and Maumy-Bertrand, 2021) and is of particular interest with big data since they turn fitting Cox models to the whole dataset into computing the null deviance residuals and then fitting to those residuals a regular PLS regression model for which fast, scalable algorithms are known de Micheaux et al. (2019).

Moreover, following the NIPALS algorithm’s principles, weights, loadings, and PLS components are computed as regression slopes. These slopes may be computed even when there are missing data using pairwise OLS.

##### 2.3.2.2 The (DK)sPLSDR Algorithm

In (Bastien et al., 2015), we proposed an original algorithm named sPLSDR by using sparse PLS regression based on deviance residuals. This algorithm can be used on a dataset featuring missing values and was implemented in the plsRcox R package (Bertrand et al., 2014; Bastien et al., 2015; Bertrand and Maumy-Bertrand, 2021).

Kernel techniques allow working on a condensed matrix whose size is considerably smaller than the original one. Similarly, linear kernel PLS regression solves computational problems posed by large to huge matrices (Lindgren et al., 1993; Rännar et al., 1994) and non-linear kernel, in addition, find non-linear pattern in the data In (Bastien et al., 2015), we proposed an another original algorithm named DKsPLSDR by using the non-linear kernel counterpart of sPLSDR.

##### 2.3.2.3 Group and Sparse Group Extensions of (DK) (s)PLSDR Algorithm

Any flavour of sparse PLS regression may be applied to deviance residuals such as the two PLS extensions, called group PLS (gPLS) and sparse gPLS (sgPLS), that were proposed in (Liquet et al., 2015). As a consequence, we propose in this article two new algorithms gPLSDR and sgPLSDR, useful, for instance, to find biomarkers in genomics or proteomics datasets.

It is straightforward to extend this algorithm to group or sparse group PLS (Liquet et al., 2015), giving rise to DKgPLSDR or DKsgPLSDR. However, non-linear kernel (sparse) (group) PLS regression loses the explanation with the original descriptors unlike linear kernel PLS regression, which could limit the interpretation of the results.

In addition, we propose another extension of all the deviance based algorithms (PLSDR, sPLSDR, gPLSDR, sgPLRDR, and their kernel counterparts DKPLSDR, DKsPLSDR, DKgPLSDR, DKsgPLDR) to big data. First fit Cox models to the whole dataset to derive the null deviance residuals. Then fit to those residuals a regular, sparse, group or even sparse group PLS regression model for which fast, scalable algorithms are known de Micheaux et al. (2019).

**Algorithm 1:** The (DK)(s)(g)PLSDR algorithm

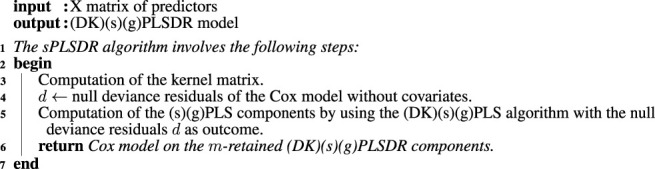

## 3 Simulation Studies

### 3.1 Scheme of the Studies

Our two in silico studies aim twofold: evaluating the accuracy of the cross-validation methods, see Section 4, and revisit the performance of the component-based methods, see Section 5.

We performed a simulation study (Algorithm 2) to benchmark the methods. For all three different simulation types [cluster by Bair et al. (2006), factorial by Kaiser and Dickman (1962) and Fan et al. (2002) or eigengene by Langfelder et al. (2013)], we simulated 100 datasets with exponential survival distribution and 40% censored rate (100 observations × 1,000 genes). We applied either no link or a linear one between the response and the predictors.

We wanted to abide by the 2:1 scheme of Bøvelstad et al. (2007); van Wieringen et al. (2009); Lambert-Lacroix and Letué (2011) and the 9:1 scheme of Li (2006). Hence, we divided each of these 600 datasets into a training set of 7/10 (70) of the observations used for estimation and a test set of 3/10 (30) of the observations used to evaluate or test the prediction capability of the estimated model.

We balanced, both according to the response value and censor rate, the division between training and test sets using the caret R package, Kuhn (2014).

### 3.2 Data Generation

#### 3.2.1 Eigengene

Given module seeds and a desired size for the genes modules around the seeds of *n*_*I*_ genes, module genes expression profiles are generated such that the *k*th rank correlated gene from module *I* with its module seed *seed*_*I*_ is:

$$\mathrm{cor}\left(x_{k,I},seed_{I}\right)=1-k/n_{I}\left(1-r_{\text{min}}\right)=r_{k,I}$$ (6)

that is, the first gene has correlation *r*_*i*,*I*_ ≈ 1 with the seed while the last (*n*_*I*_-th) gene has correlation $r_{n_{i},I}\approx r_{\text{min}}$ .

The required correlation (6) is achieved by calculating the *k*th gene profile as the sum of the seed vector *seed*_*I*_ and a noise term *a*_*k*_ *ɛ*_*k*_

$$x_{k,I}=seed_{I}+a_{k}\varepsilon_{k}\;\text{where}\;a_{k}=\sqrt{\frac{\mathrm{var}\left({seed}_{I}^{\;}\right)}{\mathrm{var}\left(\varepsilon_{k}\right)}\left(\frac{1}{r_{k,I}^{2}}-1\right)}$$ (7)

This technique produces modules consisting of genes distributed symmetrically around the module seed; in this sense, the simulated modules are spherical clusters whose centres coincide (on average) with the module seed (Langfelder et al. 2013).

**Algorithm 2:** Summary of the procedure for evaluating the accuracy of the cross validation methods and revisit the performance of the component based methods.

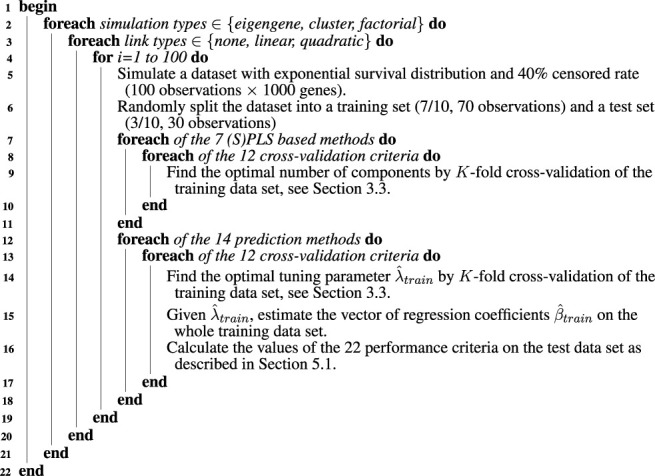

In the simulations the parameters have been let as follow *I* = 4, *r*_min_ = 0.5, *n*_*I*_ = 25 with *seed*_*I*_ and $\varepsilon_{k}∼N\left(0,1\right)$ .

Survival and censoring times, with 0.4 censoring probability, are generated from exponential survival distributions. When linked to survival (linear or quadratic case), only expressions from genes from the first two modules (*N* = 50) are related to survival time.

Each simulated data set consists of 1,000 genes and 100 samples. Only the first hundred genes are structured. The last 900 are random noise generated from N(0,1) .

#### 3.2.2 Cluster

In Bair et al. (2006) the gene expression data is distributed as:

$$X_{ij}=\left\{\begin{array}{c} 3+\varepsilon_{ij}^{\;}\text{ if }i\leq 50,j\leq 50\\ 4+\varepsilon_{ij}^{\;}\text{ if }i>50,j\leq 50\\ 3.5+\varepsilon_{ij}^{\;}\text{ if }j>50. \end{array}\right.$$ (8)

Where the *ɛ*_*ij*_ are drawn from a N(0,1) .

Each simulated data set consists of 1,000 genes and 100 samples. Survival and censoring times, with 0.4 censoring probability, are generated from exponential survival distributions. When linked to survival (linear or quadratic case), only expressions from genes from the first 50 genes are related to survival.

#### 3.2.3 Factorial

Kaiser and Dickman (1962), Fan et al. (2002) have supposed that gene expressions are related to 4 latent variables associated with a specific biological function. Let for each group a specified population inter-correlation pattern matrix *R*. By applying principal component factorization (PCA) to the matrix *R* and following Kaiser and Dickman, we can generate 4 multivariate normally distributed sample data with a specific correlation pattern. $Z_{\left(k\times N\right)}=F_{\left(k\times k\right)}X_{\left(k\times N\right)}^{\;}$ , where *k* is the number of descriptors (genes), *N* is the number of observations, *X* is a matrix of uncorrelated random standard variables N(0,1) , *F* is a matrix containing principal component factor pattern coefficients obtained by applying Principal Components Analysis (PCA) to the given population correlation matrix *R* and *Z* is the resultant sample data matrix as if sampled from a population with the given population correlation matrix *R*.

We have chosen a compound symmetry structure for the correlation matrix *R* with the identical correlation (0.7) between two descriptors of the same group, descriptors between different groups being independent.

Moreover, the correlation coefficient choice allows specifying the percentage of variance explained by the first factorial axes. Given four groups with an inter-genes correlation coefficient of 0.7 corresponds to expend 70*%* of the inertia in 4 principal directions.

Survival and censoring times, with 0.4 censoring probability, are generated from exponential survival distributions. When linked to survival (linear or quadratic case), only expressions from genes from the first two groups (*N* = 50) are related to survival time.

Each simulated data set consists of 1,000 genes and 100 samples. Only the first hundred genes are structured. The last 900 are random noise generated from N(0,1) .

### 3.3 Hyperparameters and Cross-Validation

First, create *K* folds of size Floor (*n*/*K*) by sampling without replacement and then assign randomly to a different fold each of the remaining *n*  mod  *K* data points. Those folds can be used to perform standard *K*-fold cross-validation of a dataset of size *n*.

To perform stratified or balanced cross-validation (Breiman et al., 1984, *p*. 246), we need first to order the data by the response value or class and then bin those values into *c* classes, each containing *K* points with many similar response values. Any extremal remaining points are assigned to an additional bin, and a fold is obtained by sampling once without replacement from each of the bins. This is the only difference between balanced cross-validation and standard cross-validation. In the simulation study, We used balanced cross-validation with respect to the response value and censor rate. The folds were design using the caret R package, Kuhn (2014).

In traditional cross-validation, *i.e.,* with a dataset without censored events, each fold would yield a test set and a value of a prediction error measure (for instance, the log partial likelihood, the integrated area under the curve, the integrated area under the prediction error curve). When dealing with censored events and using the CV partial likelihood (CVLL, Verweij and Van Houwelingen (1993)) criterion, it is possible to make more efficient use of risk sets: van Houwelingen et al. (2006) recommended to derive the CV log partial likelihood for the *j*th fold by subtraction; by subtracting the log partial likelihood evaluated on the full dataset from that evaluated on the full dataset minus the *j*th fold, called the (*K* − 1)/*K* dataset. Such a derivation of the CV log partial likelihood yields the van Houwelingen CV partial likelihood (vHCVLL).

Hyperparameters —the number of components for PLS models and their extensions and both the number of components and the thresholding parameter *η* for sparse PLS models—were tuned using 7-fold cross-validation on the training set. The number of folds was chosen following the recommendation of Wold et al. (2001), Breiman and Spector (1992) and Kohavi (1995). As in, Bøvelstad et al. (2007), van Wieringen et al. (2009) and Lambert-Lacroix and Letué (2011), mean values were then used to summarize these cross-validation criteria over the seven runs and the hyperparameters were chosen according to the best values of these measures. A special case is the autoPLS-Cox algorithm that stops adding components to the model as soon as each predictor is no longer significant in the model.

## 4 Highlighting Relevant Cross Validation Criteria

### 4.1 The Failure of the Two Usual Criteria

**FIGURE 1:**
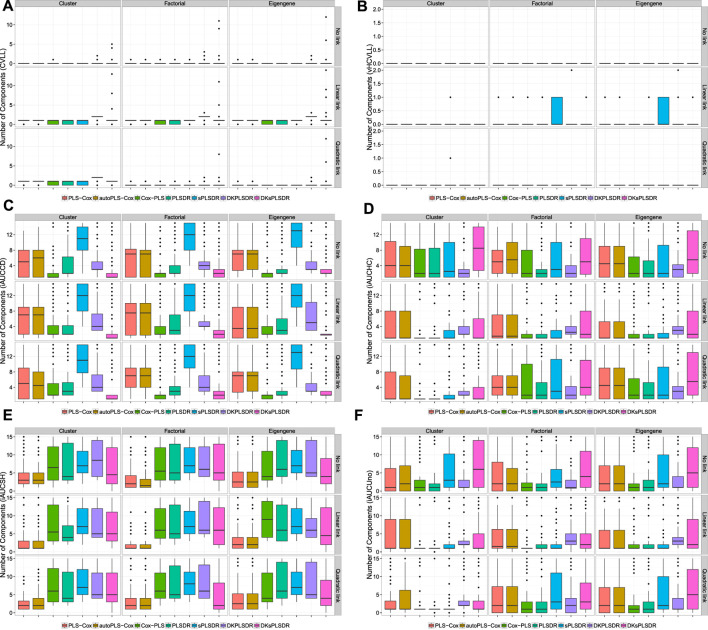
Number of components. panel **(A)**: LL criterion. panel **(B)**: vHLL criterion. panel **(C)**: iAUCCD criterion. panel **(D)**: iAUCHC criterion. panel **(E)**: iAUCSH criterion. panel **(F)**: iAUCUno criterion.

The van Houwelingen CV partial likelihood (vHCVLL, see Figure 1B) criterion behave poorly for all the PLS or sPLS based methods by selecting zero components where, according to our simulation types, the PLS-Cox, autoPLS-Cox, Cox-PLS, PLSDR, sPLSDR, DKPLSDR and DKsPLSDR methods were expected to select, for the factor or eigengene schemes, about two components and slightly more for the cluster scheme. As with the classic CV partial likelihood (CVLL), it almost always selects at most one component and systematically underestimates the number of components. Figure 1A displays the simulations results for selecting the number of components using CVLL. We confirmed this insufficient property by performing cross-validation on a simpler simulation scheme designed by Simon et al. (2011).

### 4.2 Proposal of New Criteria

**FIGURE 2:**
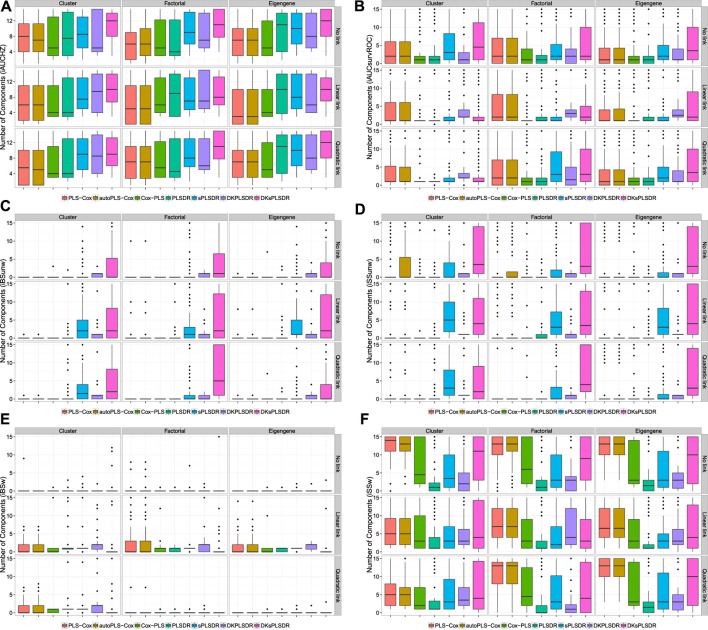
Number of components. panel **(A)**: iAUCHZ criterion. panel **(B)**: iAUCSurvROC criterion. panel **(C)**: iBSunw criterion. panel **(D)**: iSSunw criterion. panel **(E)**: iBSw criterion. panel **(F)**: iSSw criterion.

As a consequence, we had to search for other CV criteria (CVC) for the models featuring components. Li (2006) used the integrated area under the curves of time-dependent ROC curves (iAUCsurvROC, Heagerty et al. (2000)) to carry out cross-validations, implemented in the survcomp R package, (Schröder et al., 2011). Apart from that criterion (Figure 2B) we added five other integrated AUC measures: integrated Chambless and Diao’s (2006) estimator (iAUCCD, Figure 1C), integrated Hung and Chiang’s (2010) estimator (iAUCHC, Figure 1D), integrated Song and Zhou’s (2008) estimator (iAUCSH, Figure 1E), integrated Uno et al.’s (2007) estimator (iAUCUno, Figure 1F) and integrated Heagerty and Zheng’s (2005) estimator (iAUCHZ, Figure 2A) of cumulative/dynamic AUC for right-censored time-to-event data, implemented in the survAUC R package, Potapov et al. (2012), and the risksetROC R package, Heagerty and packaging by Paramita Saha-Chaudhuri (2012). We also studied two versions of two prediction error criteria, the integrated (un)weighted Brier Score (Graf et al. (1999), Gerds and Schumacher (2006), iBS(un)w, integrated (un)weighted squared deviation between predicted and observed (iAUCSH), implemented in the survAUC package survival, Figures 2C,E) and the integrated (un)weighted Schmid Score (Schmid et al. (2011), iSS(un)w, integrated (un)weighted absolute deviation between predicted and observed survival, Figures 2D,F), also implemented in the survAUC R package, Potapov et al. (2012). Additional plots of the results are available as Supplementary Material S1-S12 in the supplemental data.

### 4.3 Analysis of the Results

The simulation results highlighted the integrated Song and Zhou’s estimator of cumulative/dynamic AUC for right-censored time-to-event data (iAUCSH), implemented in the survAUC R package, Potapov et al. (2012), as the best CV criterion for the PLS-Cox and the autoPLS-Cox methods even though it behaves poorly in all the other cases.

As for the other models featuring components, the iAUCsurvROC, iAUCUno criterion exhibited the best performances. The two unweighted criteria iBSunw and iSSunw uniformly fail for all the models. The iBSw criterion is too conservative and wrongly selects null models in more than half of the cases in the linear link scheme and in almost every times in the quadratic scheme. The iSSw provides very poor results for Cox-PLS, sPLSDR and DKsPLSDR methods and average results for PLSDR and DKPLSDR methods.

The two models SPLSDR and DKSPLSDR use an additional parameter: the thresholding parameter *η*. The same figures were produced for all the criteria (Supplementary Material S13-S36 in the supplemental data): both iAUCUno criterion and iAUCsurvROC criterion provided a reasonable spread for the *η* parameter.

### 4.4 Recommendation

In a word, this simulation campaign enables us to state the following recommendations to firmly improve the selection of the right number of components: use iAUCSH to cross-validate PLS-Cox or autoPLS-Cox models and either iAUCUno or iAUCsurvROC to cross-validate Cox-PLS, PLSDR, sPLSDR, DKPLSDR and DKsPLSDR. We implemented these recommendations (iAUCSH for PLS-Cox or autoPLS-Cox models and iAUCsurvROC for Cox-PLS, PLSDR, sPLSDR, DKPLSDR and DKsPLSDR) as the default cross validation techniques in the plsRcox R package. We will apply them in the remaining of the article to assess goodness of fit of the model.

## 5 Reassessing Performance of (s)PLS Based Models

We will now provide evidence that the changes of the cross-validation criteria recommended in Section 4.4 actually lead to performance improvements for the fitted models.

### 5.1 Introduction to Performance Criteria Analysis

We followed the methodological recommendations of van Wieringen et al. (2009) to design a simulation plan that ensures a good evaluation of the predictive performance of the models.

“The true evaluation of a predictor’s performance is to be done on independent data. In the absence of independent data (the situation considered here) the predictive accuracy can be estimated as follows Dupuy and Simon (2007). The samples are split into mutually exclusive training and test sets. The gene expression and survival data of the samples in the training set are used to build the predictor. No data from the test set are used in predictor construction (including variable selection) by any of the methods compared. This predictor is considered to be representative of the predictor built on all samples (of which the training set is a subset). The test set is used to evaluate the performance of the predictor built from the training set: for each sample in the test set, survival is predicted from gene expression data. The predicted survival is then compared to the observed survival and summarized into an evaluation measure. To avoid dependency on the choice of training and test set, this procedure is repeated for multiple splits. The average of the evaluation measures resulting from each split is our estimate of the performance of the predictor built using the data from all samples.”

As to the performance criteria themselves, Schmid et al. (2011) made several points that we will take into account to carry out our performance comparison analysis.

“Evaluating the prognostic performance of prediction rules for continuous survival outcomes is an important topic of recent methodological discussion in survival analysis. The derivation of measures of prediction accuracy for survival data is not straightforward in the presence of censored observations [Kent and O’Quigley (1988); Schemper and Stare (1996); Rosthøj and Keiding (2004)]. This is mainly due to the fact that traditional performance measures for continuous outcomes [such as the mean squared error or the *R* ^2^ fraction of explained variation) lead to biased predictions if applied to censored data (Schemper and Stare (1996)].

To overcome this problem, a variety of new approaches has been suggested in the literature. These developments can be classified into three groups: “likelihood-based approaches” [Nagelkerke (1991); Xu and O’Quigley (1999); O’Quigley et al. (2005)], “ROC-based approaches” [Heagerty et al. (2000); Heagerty and Zheng (2005); Cai et al. (2006); Uno et al. (2007); Pepe et al. (2008)], and “distance-based approaches” (Korn and Simon (1990); Graf et al. (1999); Schemper and Henderson (2000); Gerds and Schumacher (2006), 2007; Schoop et al. (2008)).

When using likelihood-based approaches, the log likelihood of a prediction model is related to the corresponding log likelihood obtained from a “null model” with no covariate information. ROC-based approaches use the idea that survival outcomes can be considered as time-dependent binary variables with levels —event— and —no event— so that time-dependent misclassification rates and ROC curves can be computed for each threshold of a predictor variable of interest. If distance-based approaches are applied, a measure of prediction error is given by the distance between predicted and observed survival functions of the observations in a sample. None of these approaches has been adopted as a standard for evaluating survival predictions so far.”

To assess the goodness of fit and prediction accuracy of all the methods, we found 23 performance measures (PM) that are commonly used LRT, VarM, R2Nag, R2XO, R2OXS, iR2BSunw, iR2BSw, iRSSunw, iRSSw, iAUCCD, iAUCHC, iAUCSH, iAUCUno, IAUCHZ, iAUCSurvROC, C, UnoC, GHCI, SchemperV, iBSunw, iBSw, iSSunw, iSSw. We chose, on statistical grounds, 14 among them LRT, R2XO, iR2BSw, iRSSw, iAUCCD, iAUCHC, iAUCSH, iAUCUno, IAUCHZ, iAUCSurvROC, GHCI, SchemperV, iBSw, iSSw, and reported the results of six indices of several kind: two *R* ^2^-like measures a likelihood-based approach (LBA), R2XO, and a distance-based approach (DBA), iRSSw, a *C* index (GHCI), two *iAUC* ROC-based approaches (ROCBA), iAUCCD and iAUCSurvROC, and an integrated robust prediction error (distance-based approach, iSSw), see Table 1. The results for the remaining eight indices are similar to those shown. We now explain our process of selection of the performance criteria.

**TABLE 1:** Criteria and their use in the cross validation step or as a performance measures for assessing the quality of the model.

Criterion	Type	As a cross validation criterion			As a performance measure		
Criterion	Type	Tested	Results	Recom. For	Is a	Selected on	Results
					PM ?	statistical	
						grounds	
CVLL	LBA	**Yes**	**Yes**		No	No	No
vHCVLL	LBA	**Yes**	**Yes**		No	No	No
LRT *p*-value	LBA	No	No		**Yes**	**Yes**	No
VarM	LBA	No	No		**Yes**	No	No
R2Nag	LBA	No	No		**Yes**	No	No
R2XO	LBA	No	No		**Yes**	**Yes**	**Yes**
R2OXS	LBA	No	No		**Yes**	No	No
iR2BSunw	DBA	No	No		**Yes**	No	No
iR2BSw	DBA	No	No		**Yes**	**Yes**	No
*iRSSunw*	DBA	No	No		***New***	No	No
*iRSSw*	DBA	No	No		***New***	**Yes**	**Yes**
iAUCCD	ROCBA	**Yes**	**Yes**		**Yes**	**Yes**	**Yes**
iAUCHC	ROCBA	**Yes**	**Yes**		**Yes**	**Yes**	No
iAUCSH	ROCBA	**Yes**	**Yes**	PLS−Cox	**Yes**	**Yes**	No
				autoPLS−Cox			
iAUCUno	ROCBA	**Yes**	**Yes**	(DK) (s)PLSDR	**Yes**	**Yes**	No
				Cox−PLS			
iAUCHZ	ROCBA	**Yes**	**Yes**		**Yes**	**Yes**	No
iAUCSurvROC	ROCBA	**Yes**	**Yes**	(DK) (s)PLSDR	**Yes**	**Yes**	**Yes**
				Cox−PLS			
C	ROCBA	No	No		**Yes**	No	No
UnoC	ROCBA	No	No		**Yes**	No	Sup. Info
GHCI	ROCBA	No	No		**Yes**	**Yes**	**Yes**
SchemperV	DBA	No	No		**Yes**	**Yes**	No
iBSunw	DBA	**Yes**	**Yes**		**Yes**	No	No
iBSw	DBA	**Yes**	**Yes**		**Yes**	**Yes**	Sup. Info
iSSunw	DBA	**Yes**	**Yes**		**Yes**	No	No
iSSw	DBA	**Yes**	**Yes**		**Yes**	**Yes**	**Yes**
Total Number	25	12		12	23	14	6 (+2 SI)

### 5.2 Selection of Performance Criteria

The likelihood ratio test (LRT, Lehmann and Romano (2005)) evaluates the null hypothesis $H_{0}\;:\;\beta =0$ . Such a hypothesis means the predictors do not affect survival. The likelihood ratio test statistic is $LLR\left(\hat{\beta }\right)=-2\left(l\left(0\right)-l\left(\hat{\beta }\right)\right)$ , with *l* (.) denoting the value of the log-likelihood function. The distribution of this test statistic can be derived under the null hypothesis: it is a *χ* ^2^ distribution used to calculate the *p*-value, which summarizes the evidence against $H_{0}$ : the lower the *p*-value, the more probable that $H_{0}$ is not valid. Moreover, many others Bair and Tibshirani (2004); Bøvelstad et al. (2007); Park et al. (2002); Segal (2006) used the *p*-value of the likelihood ratio test as an evaluation measure for the predictive performance of gene expression-based predictors of survival.

In the Cox model, the variance of the martingale residuals may be used as an alternative measure of predictive performance (VarM, *cf.* section 2.1.2). In the considered setting, our findings confirmed those of van Wieringen et al. (2009): this measure cannot discriminate very well between good and poor predictors. It is therefore omitted here.

A predictor with good predictive performance should explain a high proportion of variability in the test set’s survival data. Conversely, poor predictor should explain little variability in the test set. Consequently, it would be meaningful to use the coefficient of determination (henceforth called *R* ^2^) to quantify the proportion of variability in survival data of the test set that the predictor can explain. However, the traditional definition of *R* ^2^ cannot be applied to censored data. Modified criteria have been proposed in the past: three types of likelihood-based *R* ^2^ coefficients for right-censored time-to-event data are were put forward (R2NAG, R2XO and R2OXS).

• The coefficient (R2Nag) proposed by Nagelkerke (1991):

$$R_{Nag}^{2}=1-\exp\left(-\frac{2}{n}\left(l\left(\hat{\beta }\right)-l\left(0\right)\right)\right)$$ (9)

where *l* (.) denotes the log-likelihood function.

• The coefficient (R2XO) proposed by Xu and O’Quigley (1999) that is restricted to proportional hazards regression models, because here the means of squared residuals *MSE* in the $R_{adj}^{2}$ measure for linear regression are replaced by the (weighted) sums of squared *Schoenfeld* residuals, denoted by *J*(*β*):

$$R_{XO}^{2}=1-\frac{J\left(\hat{\beta }\right)}{J\left(0\right)}\cdot$$ (10)

• The coefficient (R2OXS) proposed by O’Quigley et al. (2005) who replaced the number of observations *n* by the number of events *e*:

$$R_{OXS}^{2}\left(\hat{\beta }\right)=1-\exp\left(-\frac{2}{e}\left(l\left(\hat{\beta }\right)-l\left(0\right)\right)\right)=1-{\left(\frac{L\left(\hat{\beta }\right)}{L\left(0\right)}\right)}^{-2/e}\cdot$$ (11)

All three were implemented in the survAUC R package, Potapov et al. (2012). Others have also used these modified *R* ^2^ statistics to assess predictive performance of gene expression based predictors on survival Bair and Tibshirani (2004); Segal (2006).

Hielscher et al. (2010) carried out a comparison of the properties of these three coefficients. In a word, R2Nag is strongly influenced by censoring (negative correlation with censoring); R2OXS is less influenced by censoring and exhibits a positive correlation with censoring. From those three R2XO is the less influenced by censoring. As a consequence, we selected the R2XO as the *R* ^2^-like measure to compare the models.

The weighted Brier score *BSw*(*t*) (Brier (1950); Hothorn et al. (2004); Radespiel-Tröger et al. (2003)) is a distance-based measure of prediction error that is based on the squared deviation between survival functions. It is defined as a function of time *t* > 0 by

$$BSw\left(t\right)=\frac{1}{n}\overset{n}{\underset{i=1}{\sum }}\left[\frac{\hat{S}{\left(t|X_{i}\right)}^{2}I\left(t_{i}⩽t\wedge \delta_{i}=1\right)}{\hat{G}\left(t_{i}\right)}+\frac{{\left(1-\hat{S}\left(t|X_{i}\right)\right)}^{2}I\left(t_{i}>t\right)}{\hat{G}\left(t_{i}\right)}\right]$$ (12)

where $\hat{G}\left(.\right)$ denotes the Kaplan-Meier estimate of the censoring distribution, that is the Kaplan–Meier estimate based on the observations (*t*_*i*_, 1 − *δ*_*i*_) and *I* stands for the indicator function. The expected Brier score of a prediction model which ignores all predictor variables corresponds to the KM estimate. To derive the unweighted Brier score, *BSunw*(*t*), clear the $\hat{G}\left(t_{i}\right)$ value of the denominators.

The Schmid score *SS*(*t*) [Schmid et al. (2011)] is a distance-based measure of prediction error that is based on the absolute deviation between survival functions, instead of the squared one for the Brier-Score. It is a robust improvement over the following empirical measure of absolute deviation between survival functions that was suggested by Schemper and Henderson (2000) as a function of time *t* > 0 by:

$$SH\left(t\right)=\frac{1}{n}\overset{n}{\underset{i=1}{\sum }}\left[\frac{\hat{S}\left(t|X_{i}\right)I\left(t_{i}⩽t\wedge \delta_{i}=1\right)}{\hat{G}\left(t_{i}\right)}+\frac{\left(1-\hat{S}\left(t|X_{i}\right)\right)I\left(t_{i}>t\right)}{\hat{G}\left(t_{i}\right)}\right]$$ (13)

where $\hat{G}\left(.\right)$ denotes the Kaplan-Meier estimate of the censoring distribution which is based on the observations (*t*_*i*_, 1 − *δ*_*i*_) and *I* stands for the indicator function. With the same notations, the Schmid score is defined as a function of time *t* > 0 by:

$$SS\left(t\right)=\frac{1}{n}\overset{n}{\underset{i=1}{\sum }}|I\left(t_{i}>t\right)-\hat{S}\left(t|X_{i}\right)|\left[\frac{I\left(t_{i}⩽t\wedge \delta_{i}=1\right)}{\hat{G}\left(t_{i}^{-}\right)}+\frac{I\left(t_{i}>t\right)}{\hat{G}\left(t_{i}\right)}\right]$$ (14)

where $t_{i}^{-}$ is a survival time that is marginally smaller than *t*_*i*_. To derive the unweighted Schmid score, *SSunw*(*t*), clear the $\hat{G}\left(t_{i}^{-}\right)$ and $\hat{G}\left(t_{i}\right)$ values of the denominators.

Brier-Score lie between 0 and 1. At time *t*, good predictions result in small Brier-Scores. The squared predicted probability that individual *i* survives until time *t* if he actually died (uncensored) before *t*, or zero otherwise, is the numerator of the first summand. This probability decrease as the survival function is better estimated. The squared probability that individual *i* dies before time *t* if he was observed at least until *t*, or zero otherwise, is the numerator of the second summand. A zero weight is affected to any censored observations with survival times smaller than *t*. The Brier-score, as defined in Eq. 12, depends on *t*. Hence, it makes sense to use the integrated Brier-Score (*IBS*) given by

$$IBS=\frac{1}{\max\left(t_{i}\right)}\int_{0}^{\max\left(t_{i}\right)}BS\left(t\right)dt.$$ (15)

as a measure to evaluate the goodness of the predicted survival functions of all observations at every time *t* between 0 and max(*t*_*i*_), *i* = 1, … , *N*.

More general than the *R* ^2^ and the *p*-value criteria associated with the log-likelihood test, as well as also appropriate for prediction methods that do not involve Cox regression models, the *IBS* has become a standard evaluation measure for survival prediction methods (Hothorn et al. (2006); Schumacher et al. (2007)).

Denoting by *BS* ^0^, the Kaplan-Meier estimator based on the *t*_*i*_, *δ*_*i*_, which corresponds to a prediction without covariates, we first define $R_{BS}^{2}$ for all *t* > 0:

$$R_{BS}^{2}\left(t\right)=1-\frac{BS\left(t\right)}{BS^{0}\left(t\right)}\cdot$$ (16)

Then the integrated iR2BSw, Graf et al. (1999), is defined by:

$$iR2BSw=\frac{1}{\max\left(t_{i}\right)}\int_{0}^{\max\left(t_{i}\right)}R_{BS}^{2}\left(t\right)dt.$$ (17)

This criterion has already been used in Bøvelstad et al. (2007) and Lambert-Lacroix and Letué (2011). The integrated iR2BSw is slightly influenced by censoring, Hielscher et al. (2010), and, as a measure based on the quadratic norm, not robust.

As a consequence, we propose and use a similar measure based on the Schmid score, the integrated R Schmid Score weighted (iRSSw), by turning the traditional *R* ^2^, derived from the quadratic norm, into the R coefficient of determination for least absolute deviation, introduced by McKean and Sievers (1987). Denoting by *SS* ^0^ the Schmid score which corresponds to a prediction without covariates, we first define *R*_*SS*_ for all *t* > 0:

$$R_{SS}\left(t\right)=1-\frac{SS\left(t\right)}{SS^{0}\left(t\right)}\cdot$$ (18)

Then the integrated iRSSw, is defined by:

$$iRSSw=\frac{1}{\max\left(t_{i}\right)}\int_{0}^{\max\left(t_{i}\right)}R_{SS}\left(t\right)dt.$$ (19)

The most widely used measure of predictive accuracy for censored data regression models is the C-index. It provides a global assessment of a fitted survival model for the continuous event time rather than focuses on the prediction of t-year survival for a fixed time. The C-index is a rank-correlation measure that aims to quantify the correlation between the ranked predicted and observed survival times by estimating the probability of concordance between predicted and observed responses. Consequently, a value of 0.5 indicates no predictive discrimination, whereas a value of 1.0 indicates a perfect separation of patients with different outcomes.

A popular nonparametric C-statistic for estimating was proposed by Harrell et al. (1996). It is computed by forming all pairs {(*y*_*i*_, *x*_*i*_, *δ*_*i*_) (*y*_*j*_, *x*_*j*_, *δ*_*j*_)} of the observed data, where the smaller follow-up time is a failure time and defined as:

$$c=\frac{\sum_{1⩽i<j⩽n}I\left(y_{i}<y_{j}\right)I\left(\hat{\beta }\prime X_{i}>\hat{\beta }\prime X_{j}\right)I\left(\delta_{i}=1\right)+I\left(y_{j}<y_{i}\right)I\left(\hat{\beta }\prime X_{j}>\hat{\beta }\prime X_{i}\right)I\left(\delta_{j}=1\right)}{\sum_{1⩽i<j⩽n}I\left(y_{i}<y_{j}\right)I\left(\delta_{i}=1\right)+I\left(y_{j}<y_{i}\right)I\left(\delta_{j}=1\right)}$$ (20)

We used the improved version (GHCI) by Gönen and Heller (2005) for the Cox proportional hazards models as a performance comparison criterion. Their estimator $K_{n}\left(\hat{\beta }\right)$ only uses the regression parameters, and the covariate distribution discarding the observed event and censoring times. For this reason, unlike Harrell’s C-index based on informative pairs, it is asymptotically unbiased. The GHCI helps to view the concordance probability as a measure of discriminatory power within the Cox model framework. This formulation provides an easy to compute and stable estimator of predictive accuracy:

$$K_{n}\left(\hat{\beta }\right)=\frac{2}{n\left(n-1\right)}\underset{1⩽i<j⩽n}{\sum }\left\{\frac{I\left(\hat{\beta }\prime \left(X_{j}-X_{i}\right)<0\right)}{1+\exp\left(\hat{\beta }\prime \left(X_{j}-X_{i}\right)\right)}+\frac{I\left(\hat{\beta }\prime \left(X_{i}-X_{j}\right)<0\right)}{1+\exp\left(\hat{\beta }\prime \left(X_{i}-X_{j}\right)\right)}\right\}\cdot$$ (21)

The partial likelihood estimator $\hat{\beta }$ mediates the effect of the observed times on $K_{n}\left(\hat{\beta }\right)$ , which is not the case for Harrell’s C-index. Besides, since the effect of censoring on the bias of $\hat{\beta }$ is negligible, the measure is robust to censoring. The coefficient features an additional property of invariance: $K_{n}\left(\hat{\beta }\right)$ remains invariant under monotone transformations of the survival times.

### 5.3 Ranking the Performance of the CV Criteria

We stated several recommendations, in Section 4 based of the accuracy of the selection of the number of components. Selecting the right number of components is a goal *per se*.

Moreover, these recommendations are also relevant from a performance criteria point of view (see Section 5.1) as the following analysis showed.

1. For all the models and simulation types, we carried out the cross-validation according to all of the 12 criteria and, for each of these criteria, we derived the value of all the 14 performance measures.
2. In order to lay the stress on the improvements of performance made when switching from the classic and the van Houwelingen log likelihood cross validation techniques to the recommended ones, we computed, for every datasets and models, all the paired differences between CVLL or vHCVLL and the eleven other CV techniques.

• Paired comparison with CVLL. For every simulated dataset we evaluated: Delta = Performance Measure (with any CV criteria ≠ CVLL) − Performance Measure (with CVLL).

• Paired comparison with vHCVLL. For every simulated dataset we evaluated: Delta = Performance Measure (with any CV criteria ≠ vHCVLL) − Performance Measure (with vHCVLL).

An analysis of these results showed a steady advantage of the recommended criteria versus either CVLL or vHCVLL especially in the linear and quadratic cases.

**FIGURE 3:**
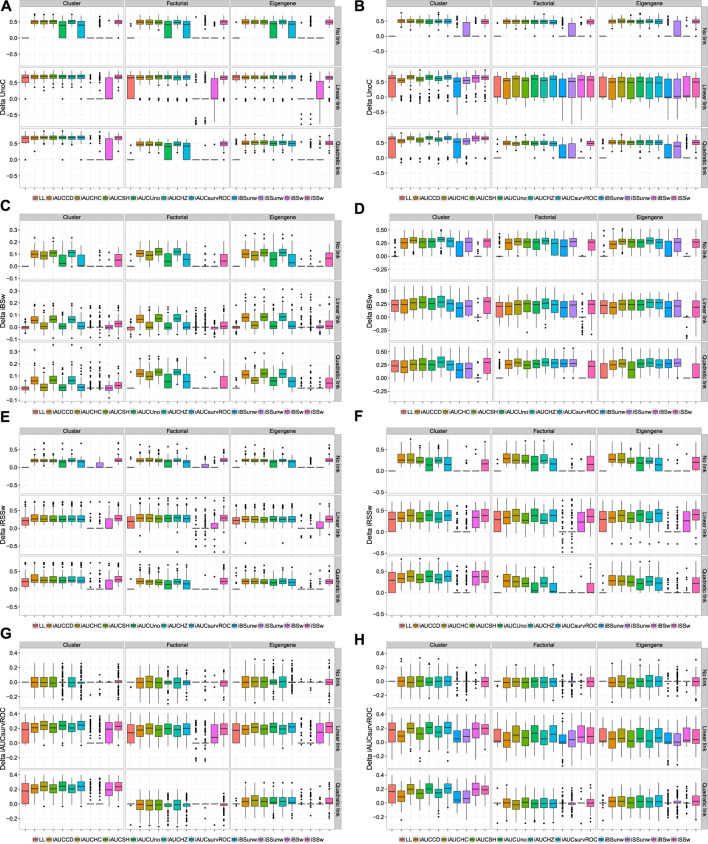
Panel by performance measure (row) and models (columns) displaying the deltas between the performance achieved for any CV crit **−** the performance achieved for vHCVLL. Top: Δ of UnoC. panel **(A)**: PLS−Cox. panel **(B)**: sPLSDR. Row 2: Δ of iBSW. panel **(C)**: PLSDR. panel **(D)**: DKsPLSDR. Row 3: Δ of iRSSW. panel **(E)**: autoPLS−Cox. panel **(F)**: PLSDR. Bottom: Δ of iAUCSurvROC. panel **(G)**: PLSDR. panel **(H)**: sPLSDR.

In the case of paired comparison with vHCVLL and for some couples of the type (performance measure, model), namely (UnoC, PLS−Cox) (UnoC, sPLSDR) (iBSW, PLSDR) (iBSW, DKsPLSDR) (iRSSW, autoPLS−Cox) (iRSSW, PLSDR) (iAUCSurvROC, PLSDR) and (iAUCSurvROC, sPLSDR), those deltas are plotted on Figures 3A-H. Additional results are available as raw results for criteria are displayed on Supplementary Material S37-S56 and deltas for paired comparisons on Supplementary Material S57-S76.

### 5.4 Performance Comparison Revisited

#### 5.4.1 Selection of Competing Benchmark Methods

Simon et al. (2011), introduced the coxnet procedure, which is an elastic net-type procedure for the Cox model, in a similar but not equivalent way than two competing ones: coxpath (glmpath R package, Park and Hastie, 2007) and penalized (penalized R package, Goeman, 2010). In Section 3 of the same article, these authors extensively compared coxnet to coxpath and to penalized for the lasso penalty that is the only one relevant for these comparisons since the three procedures use different elastic net penalties. Their results show tremendous timing advantage for coxnet over the two other procedures. The coxnet procedure was integrated in the glmnet R package (Friedman et al., 2010) and is called in the R language by applying the glmnet function with the option family = cox: coxnet is glmnet for the Cox model. The timing results of Simon et al. (2011) on both simulated and real datasets show some advantage to coxpath over penalized.

As to pure lasso-type penalty algorithms, we selected two of them: “Univariate Shrinkage in the Cox Model for High Dimensional data” (uniCox, Tibshirani, 2009) and “Gradient Lasso for Cox Proportional Hazards Model” (glcoxph, Sohn et al., 2009).

The uniCox R package implements “Univariate Shrinkage in the Cox Model for High Dimensional data” (Tibshirani, 2009). Being “essentially univariate”, it differs from applying a classical lasso penalty when fitting the Cox model and hence from both coxnet/glmnet and coxpath/glmpath. It can be used on highly correlated and even rectangular datatsets.

In their article, Sohn et al. (2009), show that the glcoxph R package is very competitive compared with popular existing methods coxpath by Park and Hastie (2007) and penalized by Goeman (2010) in its computational time, prediction and selectivity. As a very competitive procedure to coxpath, that we included in our benchmarks, and since no comparisons were carried out with coxnet, we selected glcoxph as well.

Cross validation criteria were recommended for several of our benchmark methods by their authors. We followed these recommendations —classic CV partial likelihood for coxpath, glcoxph and uniCox; van Houwelingen CV partial likelihood for coxnet with both the *λ*_*min*_, the value of *λ* that gives minimum of the mean cross-validated error, or *λ*_1*se*_, the largest value of *λ* such that the cross-validated error is within 1 standard error of the minimum of the mean cross-validated error, criteria— and used the same 7 folds fo the training set as those described in Section 3.3 for the other models.

It seemed unfair to compare the methods using a performance measure that is recommended as a cross-validation criterion for some, but not all, of them. Hence, we decided not to use any of the three recommended cross-validation criteria iAUCSH, iAUCUno or iAUCsurvROC —even if it has already been used by Li (2006)- as a performance measure, in order to strive to perform fair comparisons with the methods that are recommended to be cross validated using partial likelihood with either the classic or van Houwelingen technique.

As a consequence and in order to still provide results for a ROC-based performance measure on a fair basis, we selected the Chambless and Diao’s (2006) estimator of cumulative/dynamic AUC for right-censored time-to-event data in a form restricted to Cox regression. The integral of AUC on [0, max (times)], weighted by the estimated probability density of the time-to-event outcome, defines the iAUCCD summary measure.

#### 5.4.2 Results

For coxnet, coxlars or ridgecox with both the *λ*_*min*_ or *λ*_1*se*_ CV criteria, the *λ*_*min*_ criterion yield similar yet superior results than the *λ*_1*se*_ one whose main default is to select too often no explanatory variable (a null model) for the linear or quadratic links. As a consequence, we only reported results for the former one.

**FIGURE 4:**
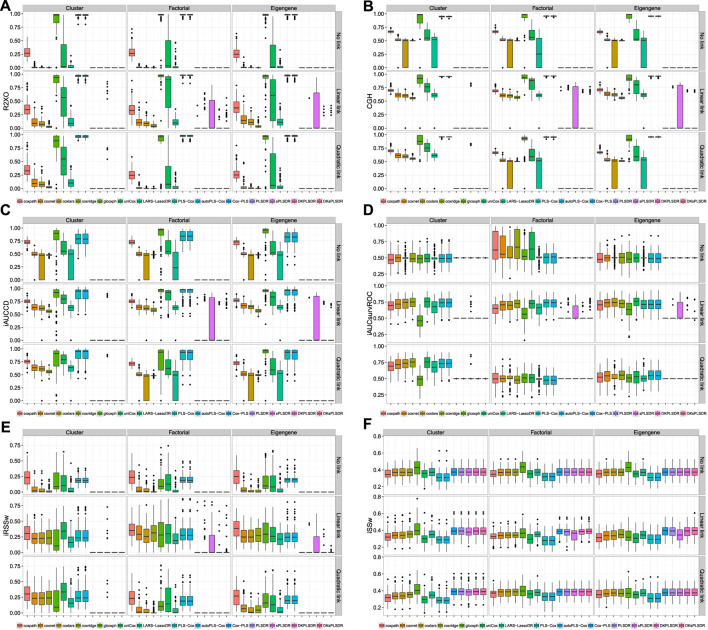
Panel by performance measure for models when fitted with vHCVLL cross validation panel **(A)**: R2XO measure. panel **(B)**: GHCI measure. panel **(C)**: iAUCCD measure. panel **(D)**: iAUCSurvROC measure, panel **(E)**: iRSSw measure. panel **(F)**: iSSw measure.

We plotted some of the performance measures when the cross-validation is done according to the vHCVLL criterion on Figures 4A-F. The results are terrible for all the (s)PLS−like models apart from PLS−Cox and autoPLS−Cox.

We then provide, for each of the (s)PLS−like method, the increases in terms of performance measures when switching from the vHCVLL as a cross validation criterion to the recommended one in Section 4.4. Virtually, for PLS-Cox and autoPLS-Cox we switch to the iAUCSH cross-validation criterion and for other (s) PLS based models to either iAUCUno or iAUCSurvROC.

**FIGURE 5:**
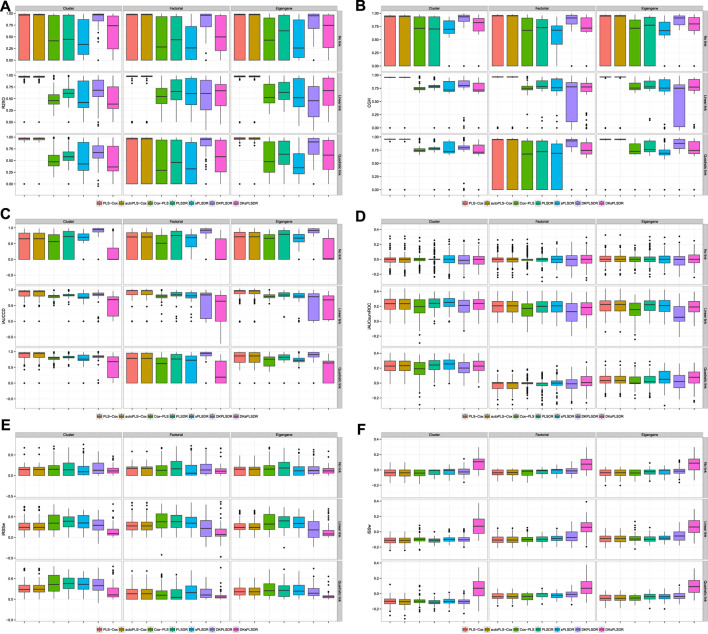
**Δ**(iAUCUno CV**−**vHCVLL), panel by measure. panel **(A)**: R2XO measure. panel **(B)**: GHCI measure. panel **(C)**: iAUCCD measure. panel **(D)**: iAUCSurvROC measure. panel **(E)**: iRSSw measure. panel **(F)**: iSSw measure.

**FIGURE 6:**
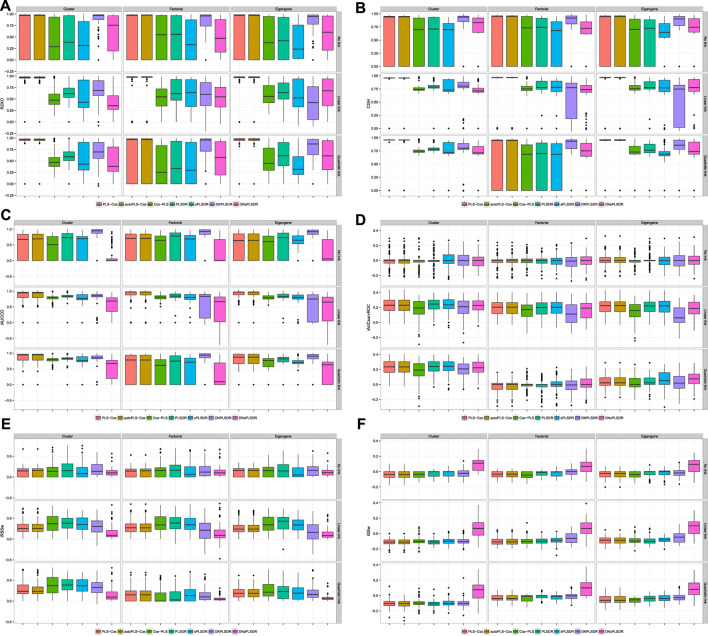
Deltas (iAUCSurvROC CV**−**vHCVLL), panel by measure. panel **(A)**: R2XO measure. panel **(B)**: GHCI measure. panel **(C)**: iAUCCD. panel **(D)**: iAUCSurvROC. panel **(E)**: iRSSw measure. panel **(F)**: iSSw measure.

For iAUCUno, these results are plotted on Figures 5A-F and whereas for iAUCSurvROC they are displayed on Figures 6A-F. These figures show a firm increase for the six criteria (R2XO, GHCI, iAUCCD, iAUCSurvROC, IRSSW, iSSW).

**FIGURE 7:**
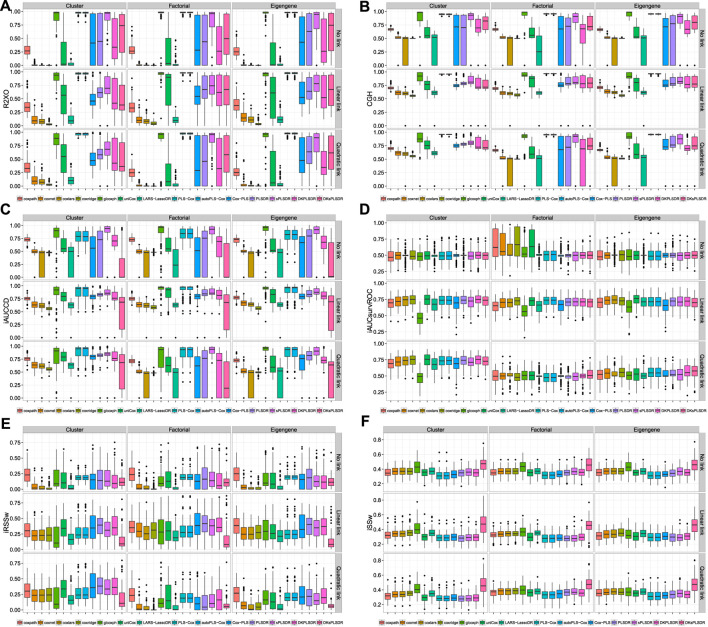
Model selected by AUCUno CV, panel by performance measure. panel **(A)**: R2XO measure. panel **(B)**: GHCI measure. panel **(C)**: iAUCCD measure. panel **(D)**: iAUCSurvROC measure. panel **(E)**: iRSSw measure. panel **(F)**: iSSw measure.

**FIGURE 8:**
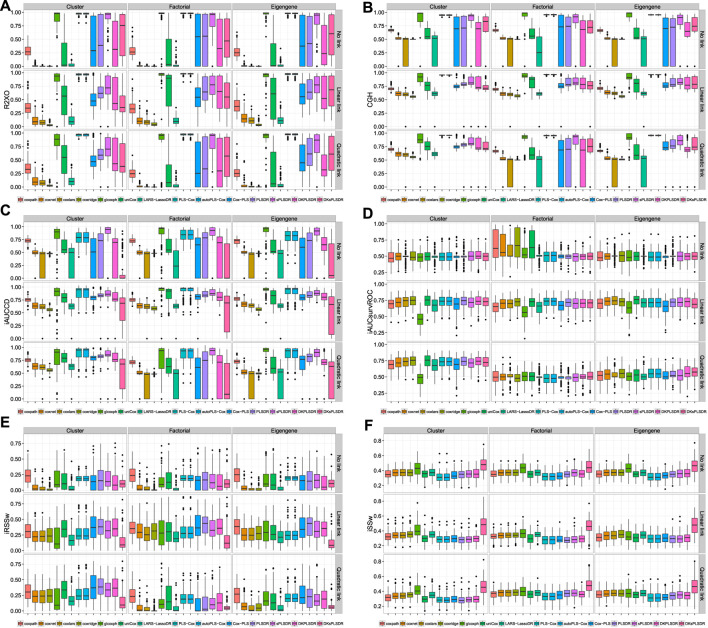
Model selected by iAUCsurvROC CV, panel by performance measure. panel **(A)**: R2XO measure. panel **(B)**: GHCI measure. panel **(C)**: iAUCCD measure. panel **(D)**: iAUCSurvROC measure. panel **(E)**: iRSSw measure. panel **(F)**: iSSw measure.

As can be seen for iAUCUno on Figures 7A-F and iAUCSurvROC on Figures 8A-F, the improvement of the performances due to switch to the recommended CV criteria is high enough to even have some (S)PLS based models, for instance SPLSDR, show some advantage over the other benchmark methods.

## 6 Conclusion

We extended our previous algorithms from Bastien et al. (2015) to enable practitioners to apply new extensions of PLS models to censored data: group and sparse group PLS regression as well as their kernel counterparts. In addition, we showed how to fit regular, sparse, group or sparse group PLS regression models and their kernel counterparts to big data. Since an interesting feature of those PLS-based extensions of Cox models is their inborn capability to cope with missing values, the partitioners can now fit survival models on censored big data with missing values.

Finding the number of components of such models is a key step in PLS models fitting. As a result we carried out a comprehensive study of cross validation criteria for those models, which lead us to an interesting result. When cross-validating standard or extended Cox models, the commonly used criterion is the cross-validated partial loglikelihood using a naive or a van Houwelingen scheme. Quite astonishingly, these two cross-validation methods fail with all the seven extensions of partial least squares regression to the Cox model, namely PLS-Cox, autoPLS-Cox, Cox-PLS, PLSDR, sPLSDR, DKPLSDR and DKsPLSDR, that we studied in Bastien et al. (2015).

In our simulation study, we introduced 12 cross validation criteria based on three different kind of model quality assessment:

• Likelihood (2): Verweij and Van Houwelingen (classic CVLL, 1993), van Houwelingen et al. (vHCVLL, 2006).
• Integrated AUC measures (6): Chambless and Diao’s (iAUCCD, 2006), Hung and Chiang’s (iAUCHC, 2010), Song and Zhou’s (iAUCSH, 2008), Uno et al.‘s (iAUCUno, 2007), Heagerty and Zheng’s (iAUCHZ, 2005), Heagerty et al.‘s (iAUCsurvROC, 2000).
• Prediction error criteria (4): integrated (un)weighted Brier Score [iBS(un)w, Gerds and Schumacher (2006)] or Schmid Score [iSS(un)w, Schmid et al. (2011)]
Our simulation study was successful in finding good CV criterion for PLS or sPLS based extensions of the Cox model:
• iAUCsh for PLS-Cox and autoPLS-Cox.
• iAUCSurvROC and iAUCUno ones for Cox-PLS (DK)PLSDR and (DK)sPLSDR.

In the presence of censored observations, the derivation of measures of prediction accuracy for survival data is not straightforward. A variety of new approaches has been suggested in the literature to overcome this problem. We spotted 23 performance measures that can be classified into three groups:

• Likelihood-based approaches (llrt, varresmart, three R2-type).
• ROC-based approaches such as integrated AUC (iAUCCD, iAUCHC, iAUCSH, iAUCUno, iAUCHZ, iAUCsurvROC), three C-index (Harrell, GHCI, UnoC).
• Distance-based approaches such as the V of Schemper and Henderson (2000) or derived from Brier or Schmid Scores (iBS(un)w, iSS(un)w and four derived R2-type measures).

Using the newly found cross-validation, and these measures of prediction accuracy, we performed a benchmark reanalysis that showed enhanced performances of these techniques and a much better behaviour even against other well known competitors such as coxnet, coxpath, uniCox and glcoxph.

Hence the recommended criteria not only improve the accuracy of the choice of the number of components but also strongly raise the performances of the models, which enables some of them to overperform the other benchmark methods.

We combined these results with the extensions to big data of our PLS based algorithms to set the cross-validation defaults in our packages.

## Data Availability

Publicly available datasets were analyzed in this study. This data can be found here: https://github.com/fbertran/Datasets_benchmark.
