# Supplementary material for: Fitting and Cross-Validating Cox Models to Censored Big Data With Missing Values Using Extensions of Partial Least Squares Regression Models
Source: Front Big Data. 2021 Nov 1;4:684794. doi: 10.3389/fdata.2021.684794 (PMC8591675; doi:10.3389/fdata.2021.684794)
Supplement: Supplementary file 1 [file DataSheet1.PDF]

Supplementary Material

1 SUPPLEMENTARY DATA

1.1 Insights on the implementation of the methods

We detail the implementation of the algorithms that we used in the article and start with some shared properties of these. Whenever the deviance residuals and survival models were to be derived, we used the `survival` R-package (Therneau and Grambsch, 2000; Therneau, 2013). As a PLS regression function in the `plsRcox` R-package, we made three wrappers using either the `pls` function of the `pls` R-package (Mevik et al., 2011), the `plsR` function of the `plsRglm` R-package (Bertrand et al., 2014; Bertrand and Maumy-Bertrand, 2021) or the `pls` function of the `mixOmics` R-package (Dejean et al., 2013). The last two are based on the NIPALS algorithm and hence automatically handle missing data (Tenenhaus, 1998) in the explanatory variables. In addition, the `pls` function of the `mixOmics` R-package (Dejean et al., 2013) can quickly handle big datasets such as Dataset 5 with 242 rows and 44754 variables. As a consequence, we had to make the `spls` function of the `spls` R-package use this function instead of the `pls` function of the `pls` R-package (Mevik et al., 2011). In addition, any flavor of sparse PLS regression may be applied to deviance residuals such as the two PLS extensions, called group PLS (gPLS) and sparse gPLS (sgPLS), that were proposed in (Liquet et al., 2015). Those flavors give rise to two new algorithms gPLSDR and sgPLSDR of particular interest, for instance to find biomarkers in genomics or proteomics datasets. Those sparse algorithms are of particular interest with big data, since they turn fitting Cox models to the whole dataset into deriving the null deviance residuals and then fitting to those residuals a sparse, group or even sparse group PLS regression model for which fast scalable algorithms are known de Micheaux et al. (2019).

1.2 Addtionnal figures

The following pages display additional simulation results to support our anaysis of cross-validation criterion.

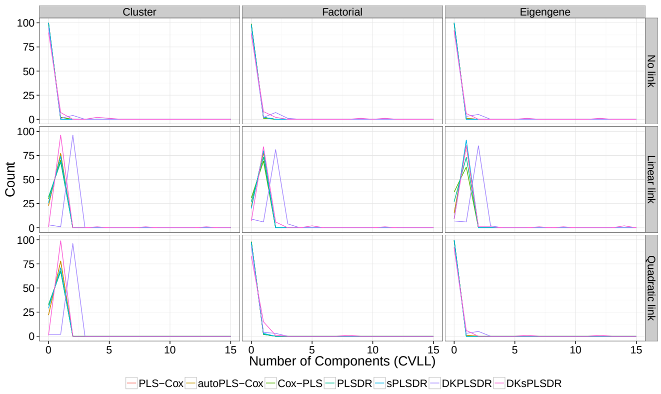

Figure S1: Nbr of comp, LL criterion.

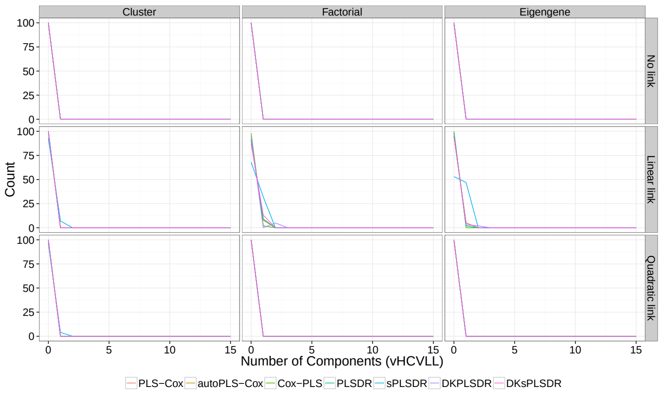

Figure S2: Nbr of comp, vHLL criterion.

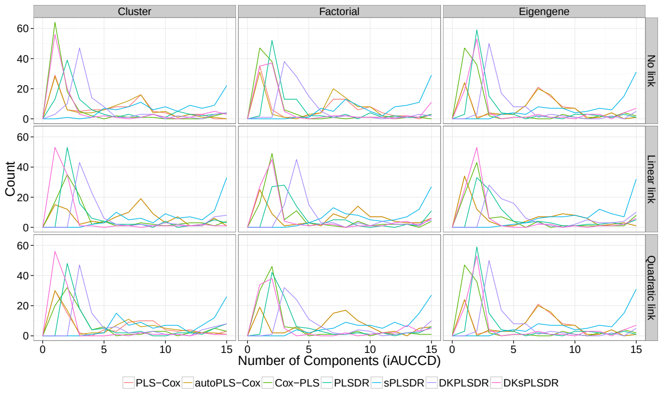

Figure S3: Nbr of comp, iAUCCD criterion.

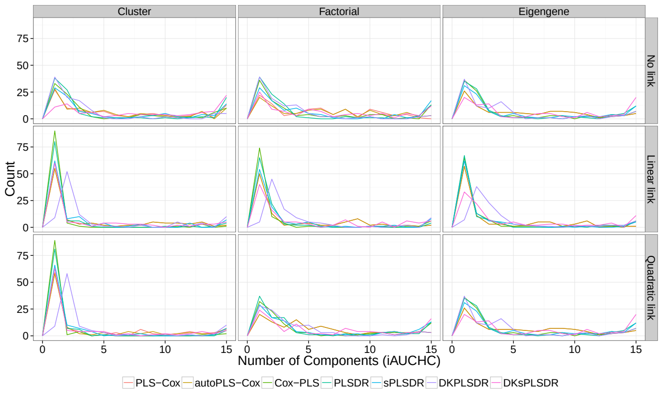

Figure S4: Nbr of comp, iAUCHC criterion.

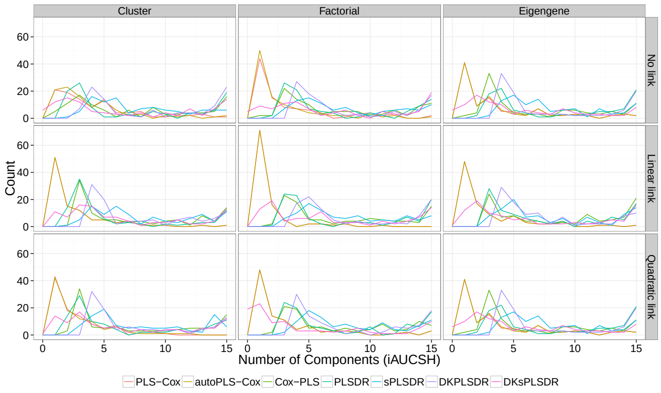

Figure S5: Nbr of comp, iAUCSH criterion.

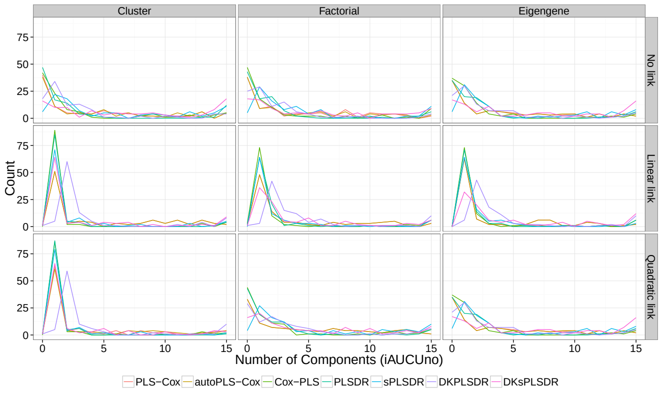

Figure S6: Nbr of comp, iAUCUno criterion.

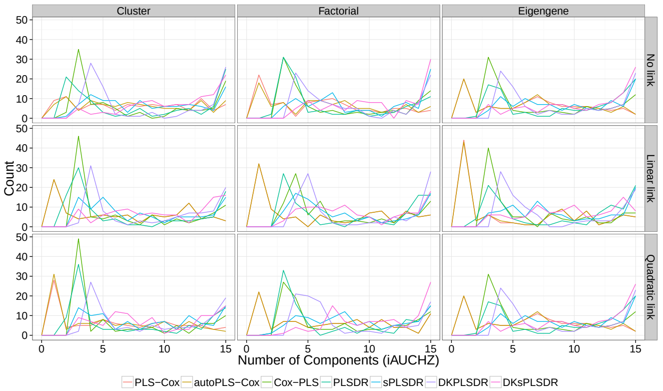

Figure S7: Nbr of comp, iAUCHZ criterion.

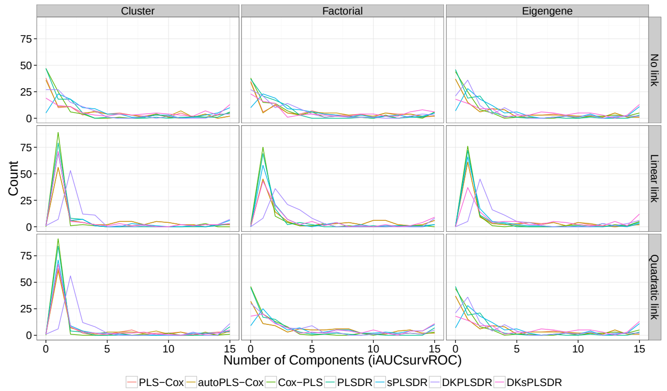

Figure S8: Nbr of comp, iAUCSurvROC criterion.

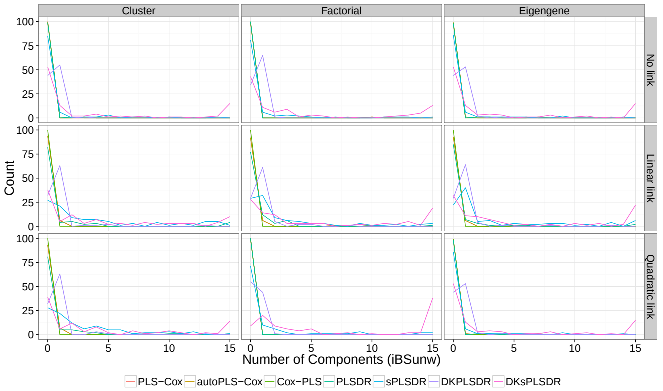

Figure S9: Nbr of comp, iBSunw criterion.

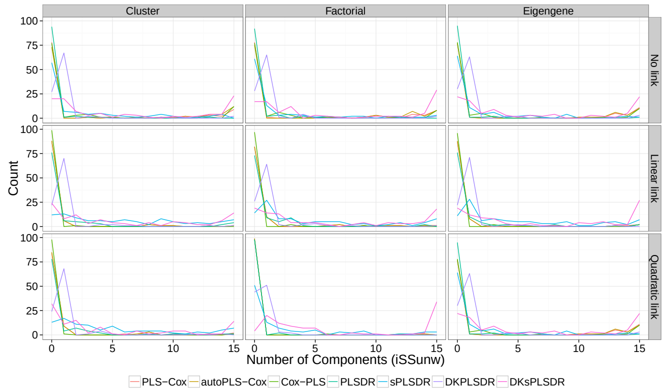

Figure S10: Nbr of comp, iSSunw criterion.

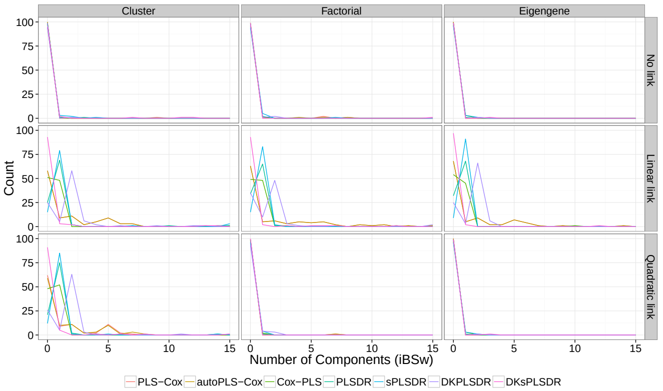

Figure S11: Nbr of comp, iBSw criterion.

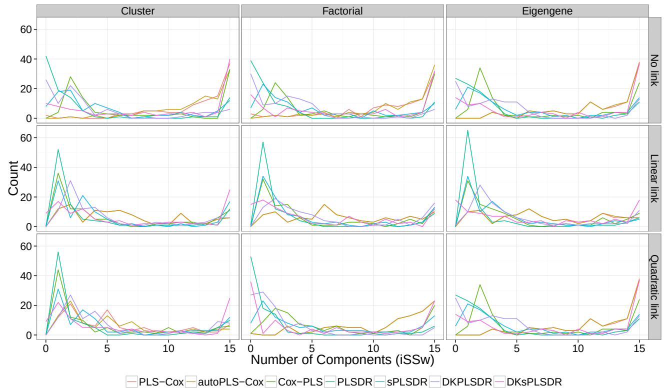

Figure S12: Nbr of comp, iSSw criterion.

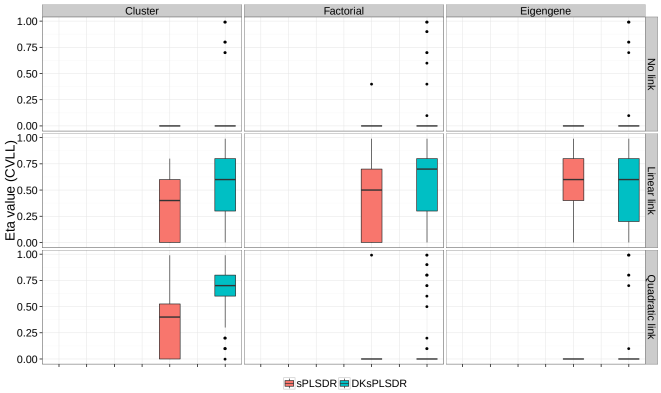

Figure S13: η, LL criterion.

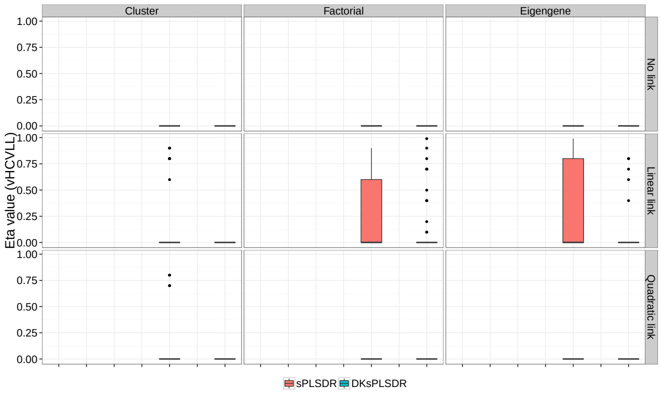

Figure S14: η, vHLL criterion.

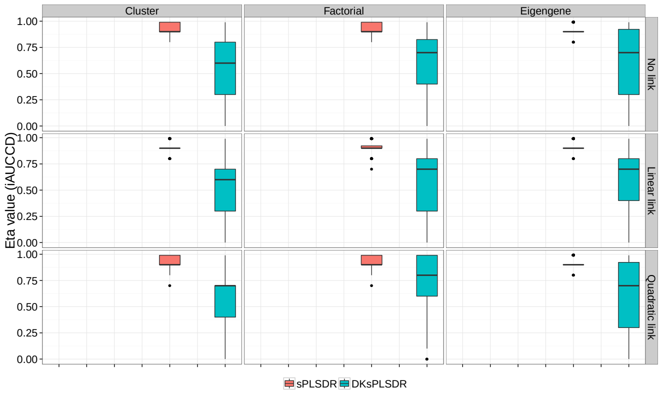

Figure S15: η, iAUCCD criterion.

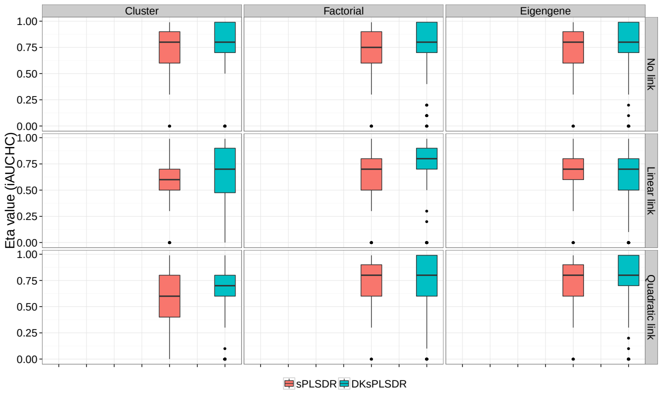

Figure S16: η, iAUCHC criterion.

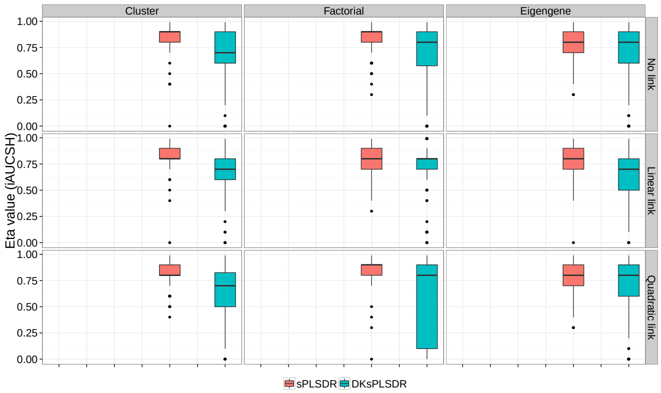

Figure S17: η, iAUCSH criterion.

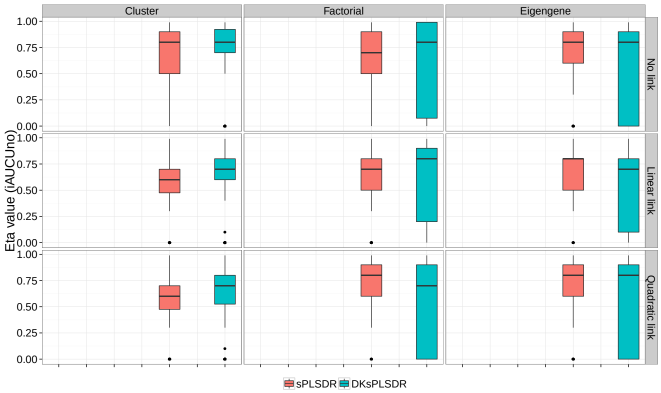

Figure S18: η, iAUCUno criterion.

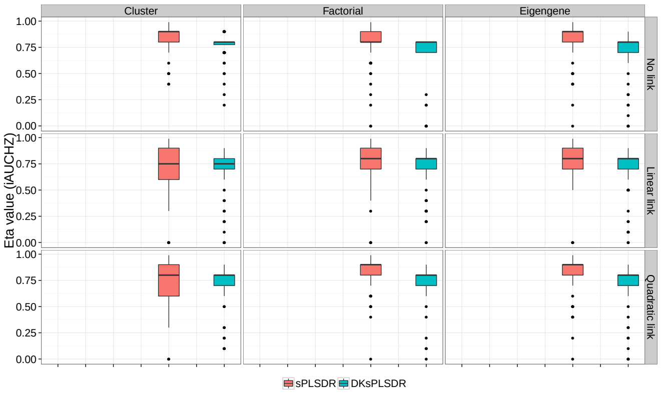

Figure S19: η, iAUCHZ criterion.

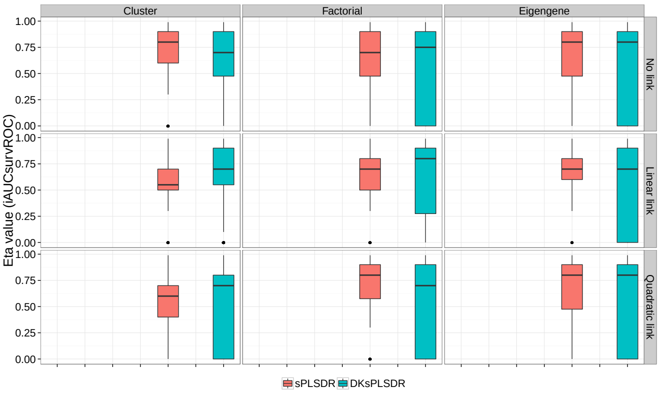

Figure S20: η, iAUCSurvROC criterion.

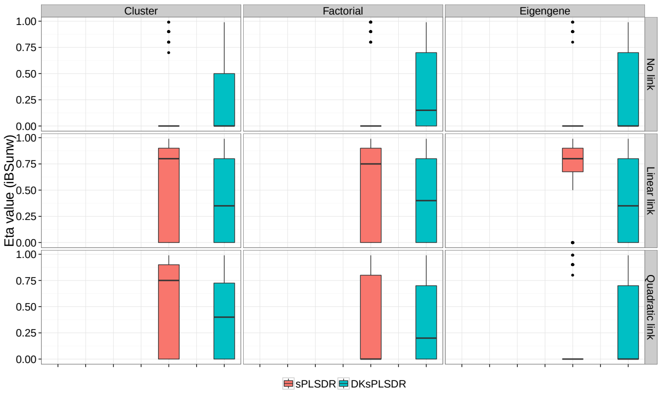

Figure S21: η, iBSunw criterion.

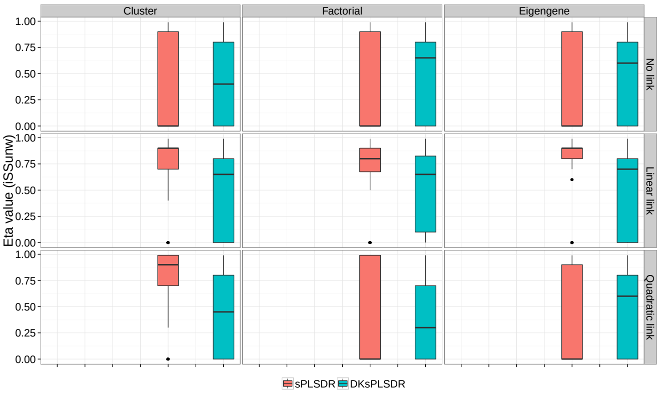

Figure S22: η, iSSunw criterion.

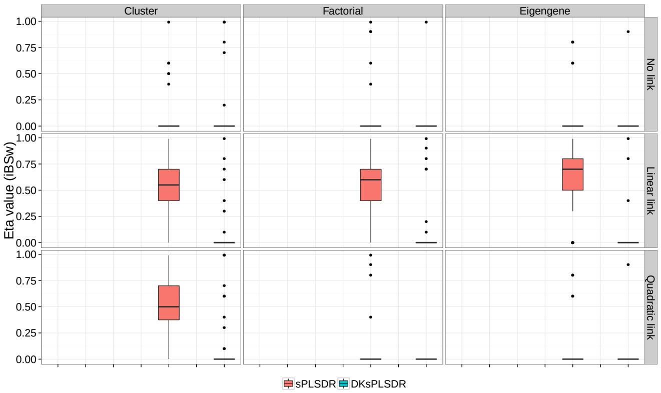

Figure S23: η, iBSw criterion.

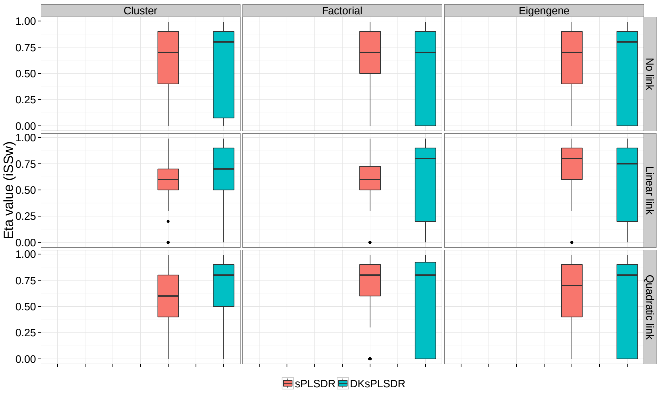

Figure S24: η, iSSw criterion.

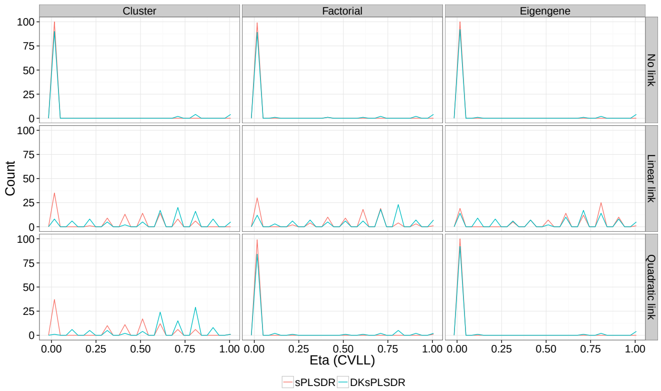

Figure S25: η, LL criterion.

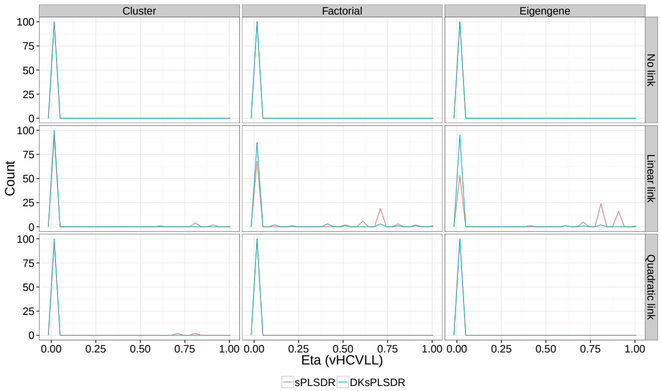

Figure S26: η, vHLL criterion.

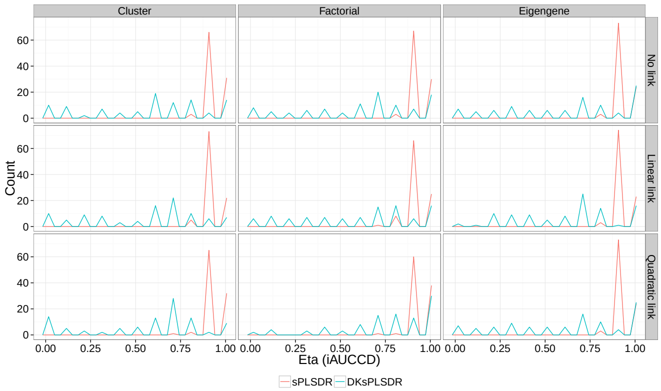

Figure S27: η, iAUCCD criterion.

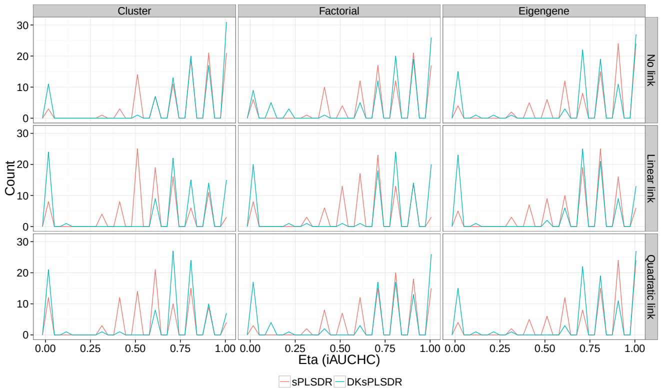

Figure S28: η, iAUCHC criterion.

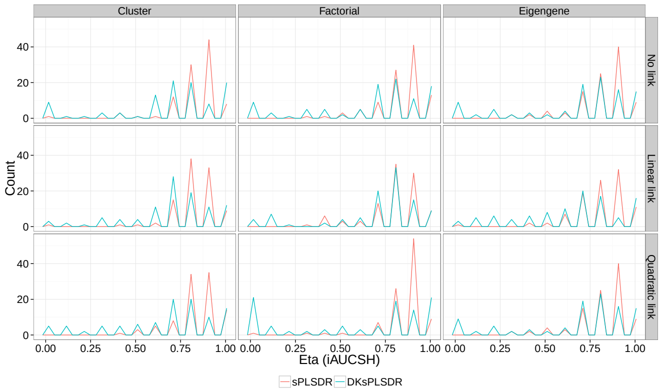

Figure S29: η, iAUCSH criterion.

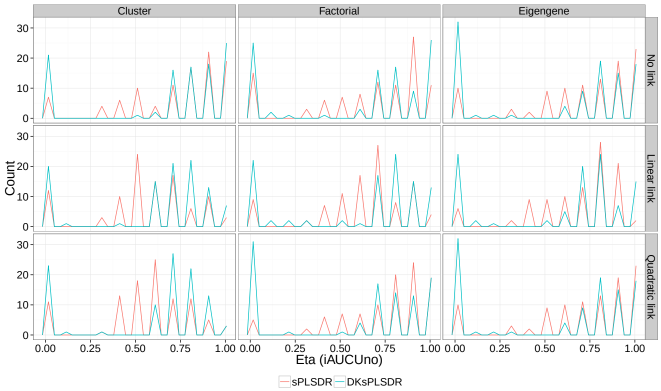

Figure S30: η, iAUCUno criterion.

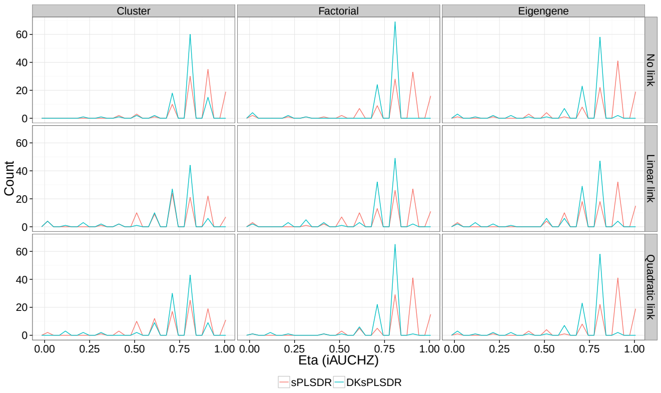

Figure S31: η, iAUCHZ criterion.

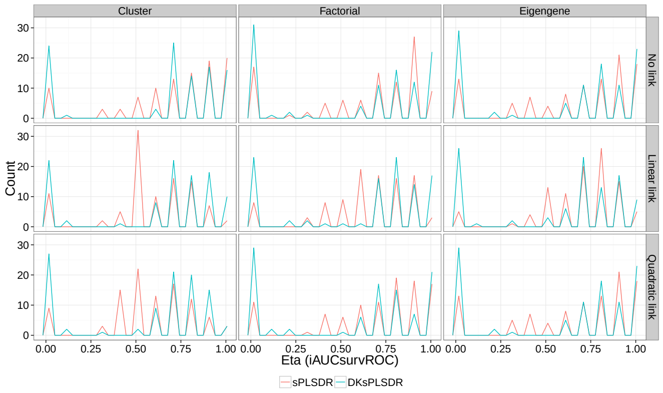

Figure S32: η, iAUCSurvROC criterion.

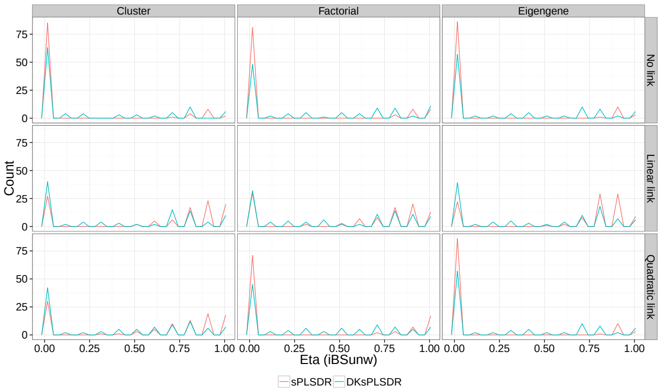

Figure S33: η, iBSunw criterion.

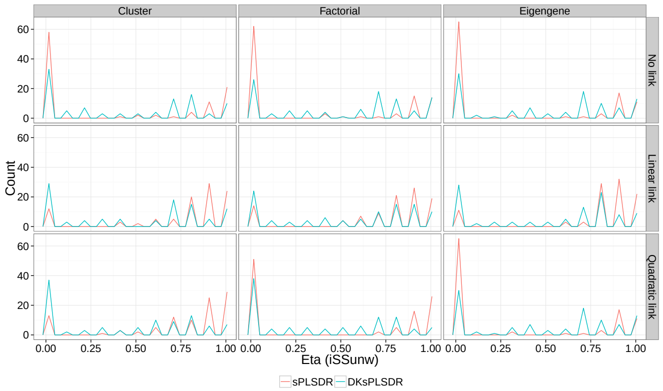

Figure S34: η, iSSunw criterion.

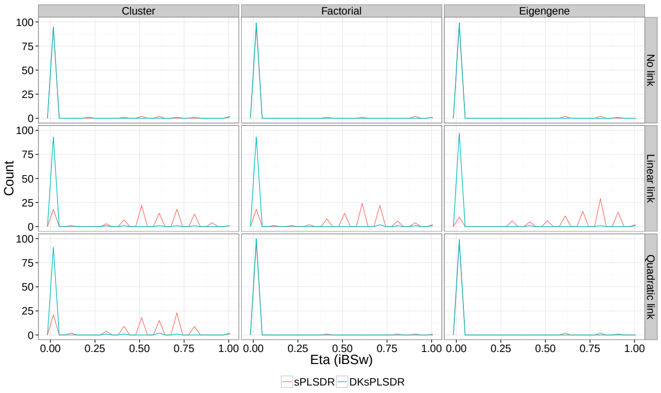

Figure S35: η, iBSw criterion.

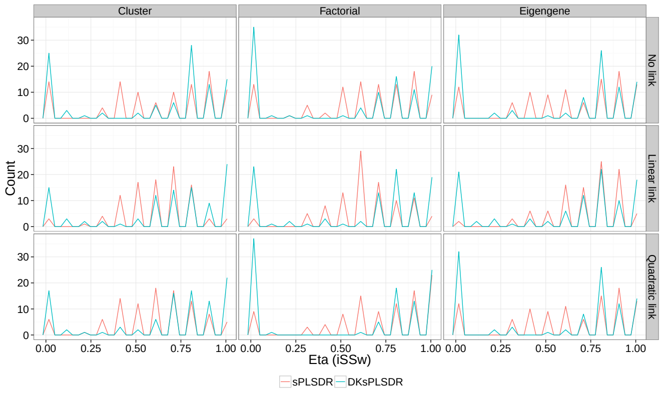

Figure S36: η, iSSw criterion.

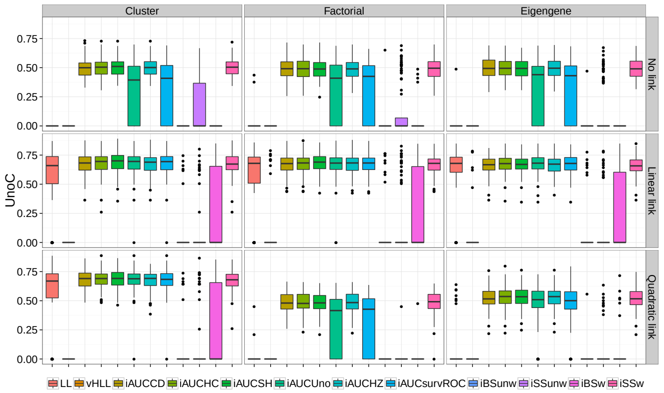

Figure S37: UnoC vs CV criterion. autoplsRcox.

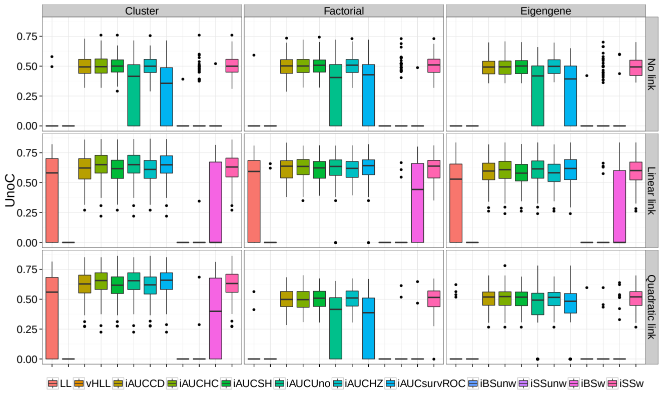

Figure S38: UnoC vs CV criterion. coxpls.

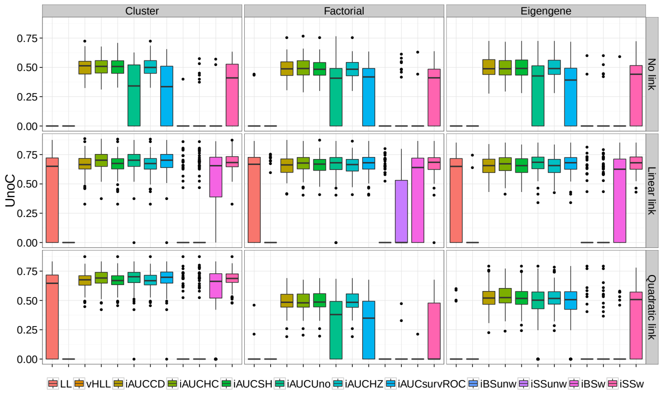

Figure S39: UnoC vs CV criterion. coxplsDR.

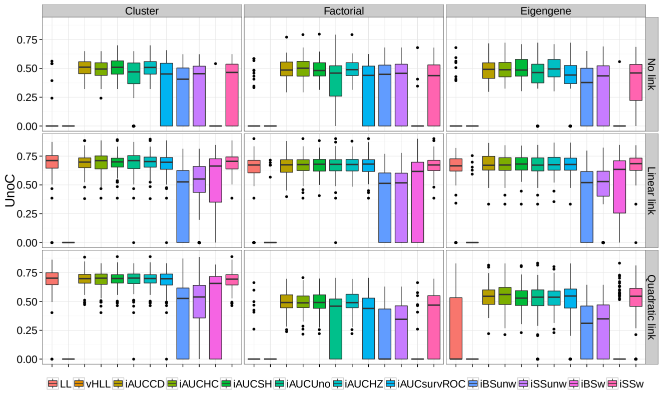

Figure S40: UnoC vs CV criterion. coxDKplsDR.

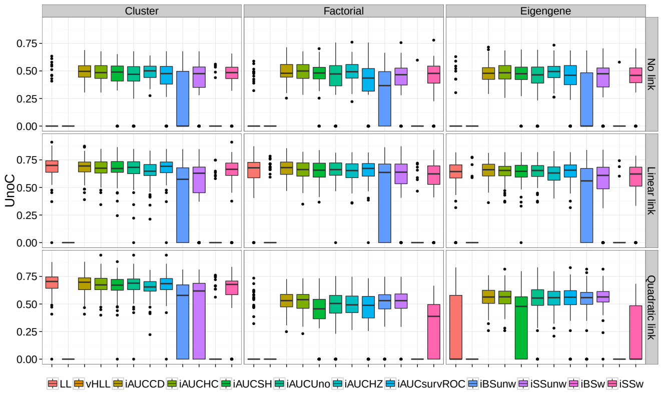

Figure S41: UnoC vs CV criterion. DKsplsDR.

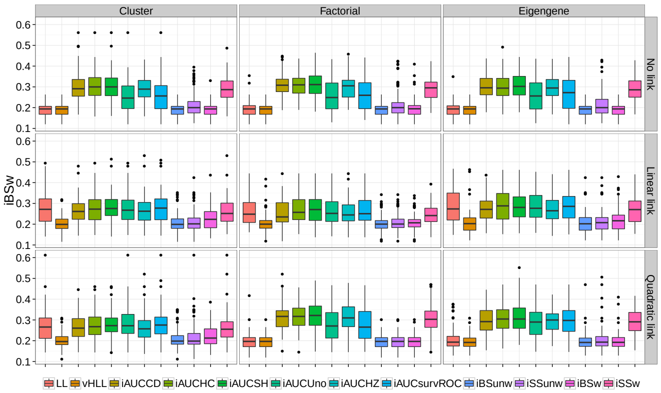

Figure S42: iBSW vs CV criterion. plsRcox.

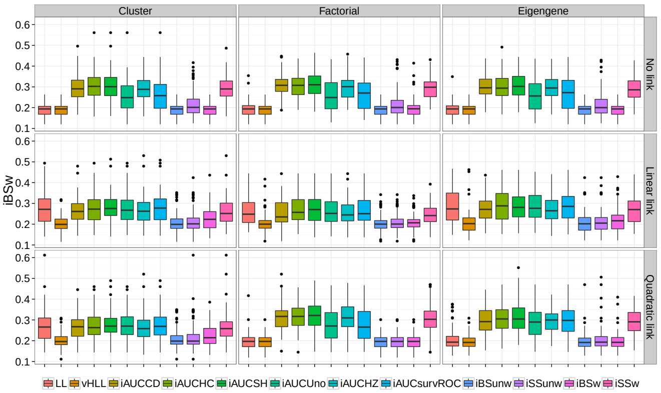

Figure S43: iBSW vs CV criterion. autoplsRcox.

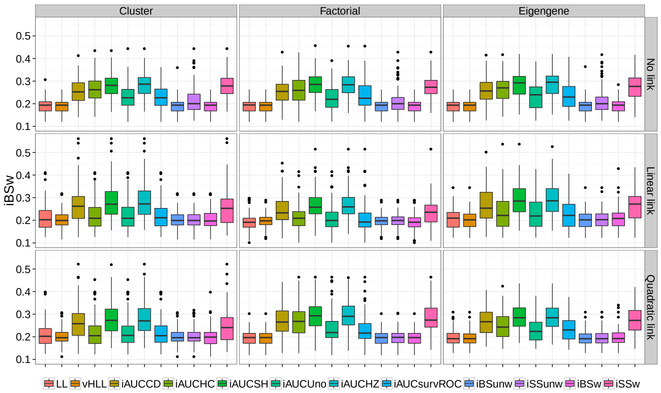

Figure S44: iBSW vs CV criterion. coxpls.

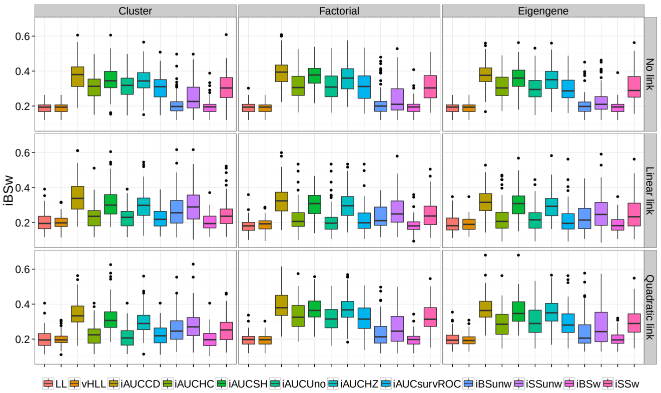

Figure S45: iBSW vs CV criterion. splsDR.

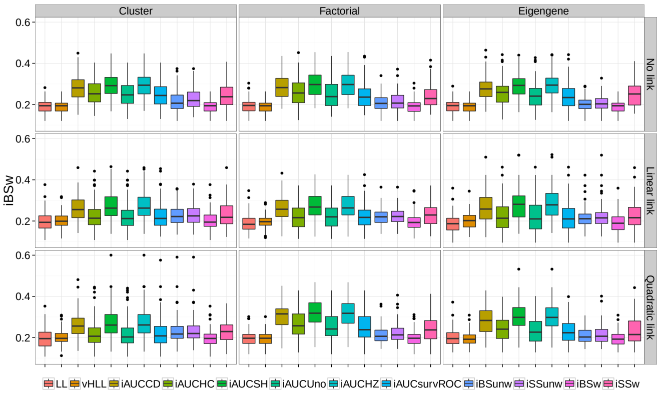

Figure S46: iBSW vs CV criterion. DKsplsDR.

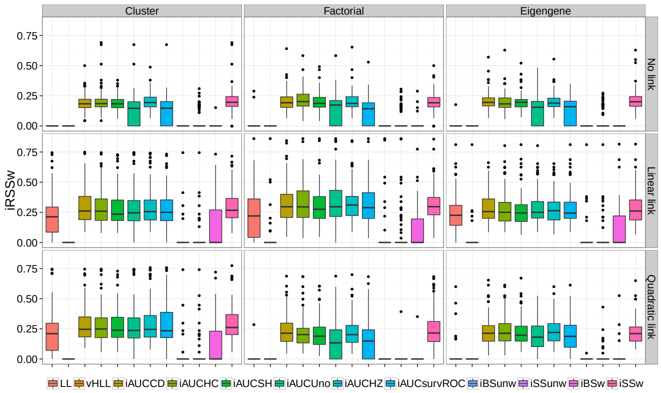

Figure S47: iRSSW vs CV criterion. plsRcox.

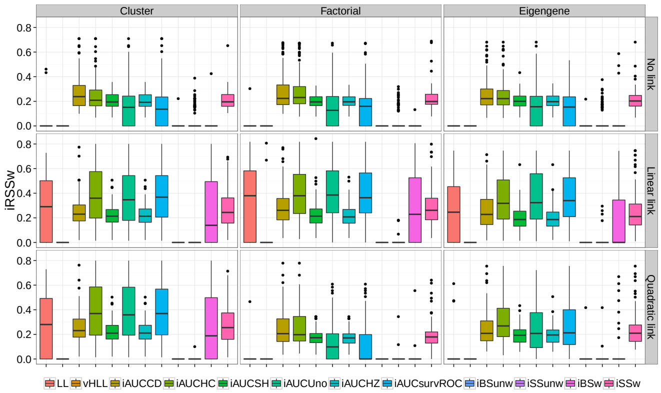

Figure S48: iRSSW vs CV criterion. coxpls.

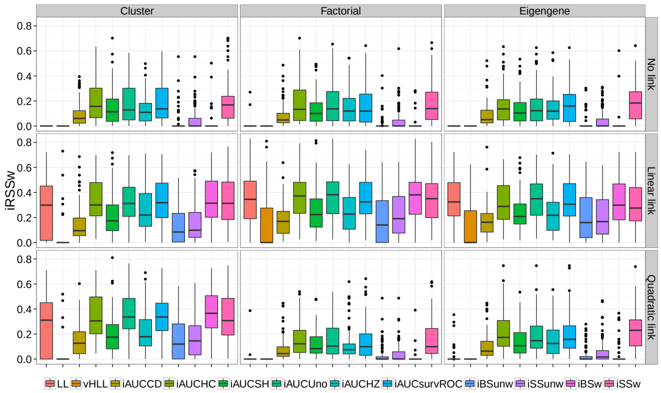

Figure S49: iRSSW vs CV criterion. coxsplsDR.

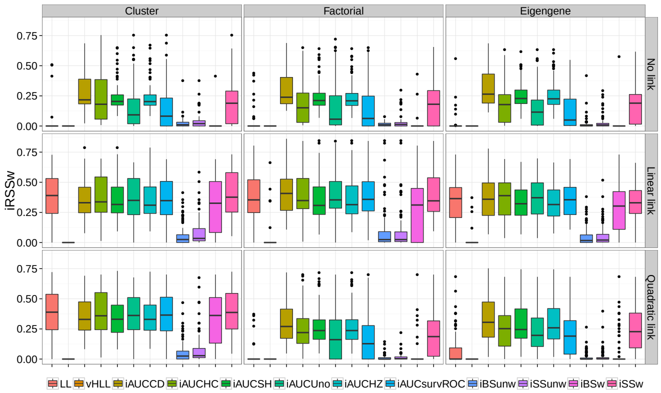

Figure S50: iRSSW vs CV criterion. DKplsDR.

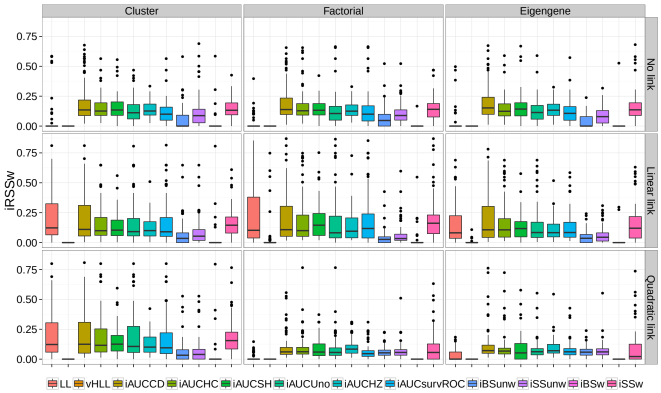

Figure S51: iRSSW vs CV criterion. DKsplsDR.

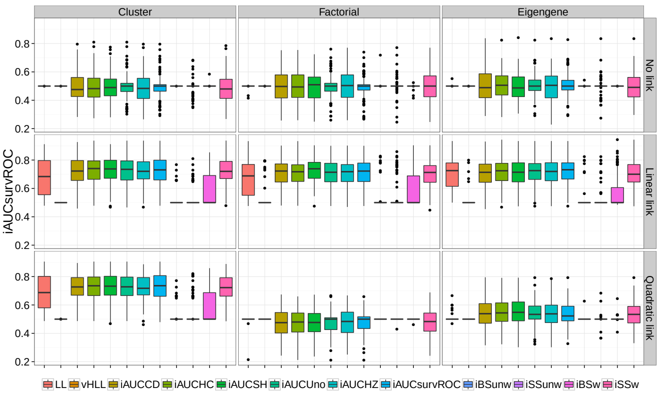

Figure S52: SurvROC vs CV criterion. plsRcox.

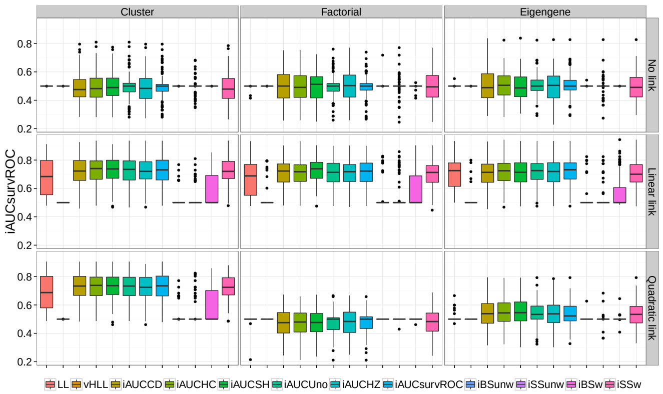

Figure S53: SurvROC vs CV criterion. autoplsRcox.

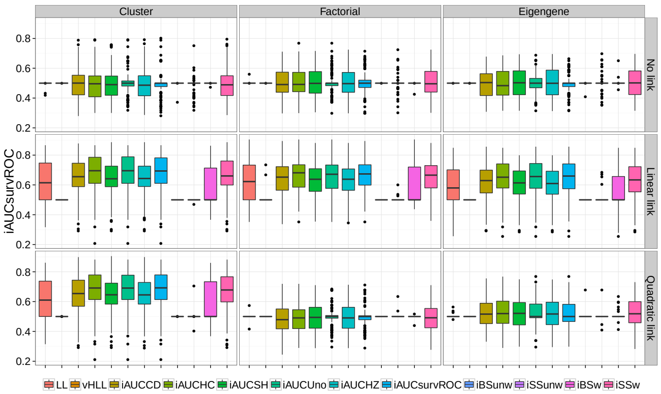

Figure S54: SurvROC vs CV criterion. coxpls.

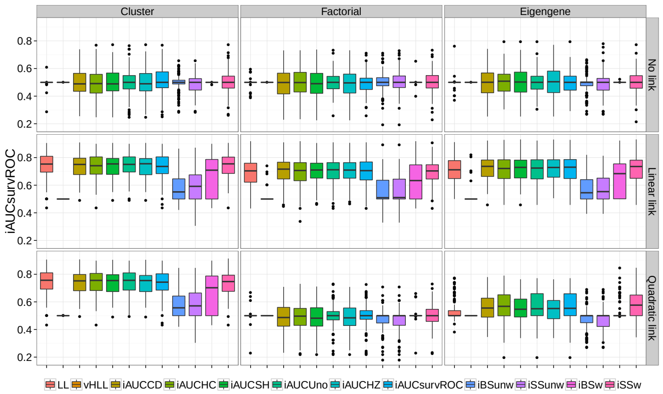

Figure S55: SurvROC vs CV criterion. DKplsDR.

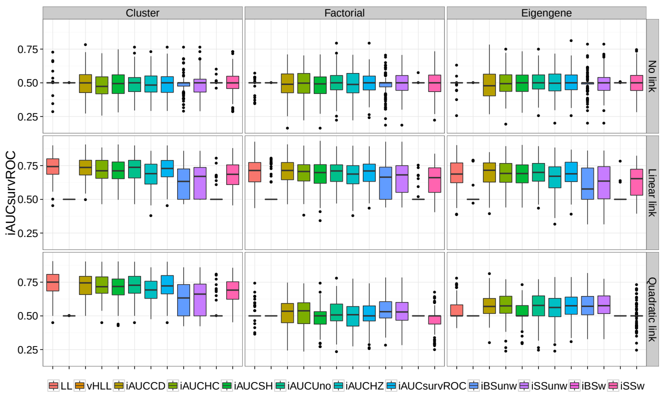

Figure S56: iAUCSurvROC vs CV criterion, DKsplsDR.

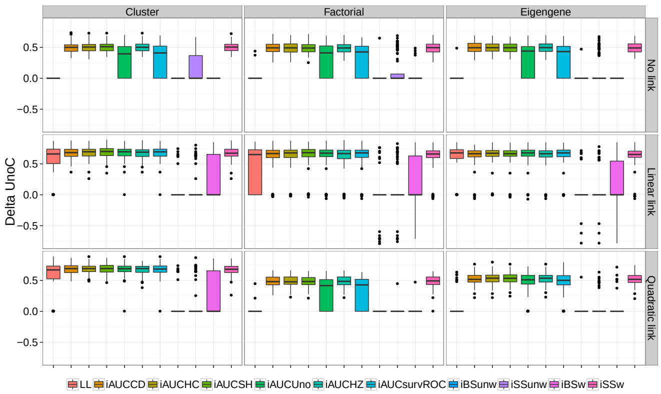

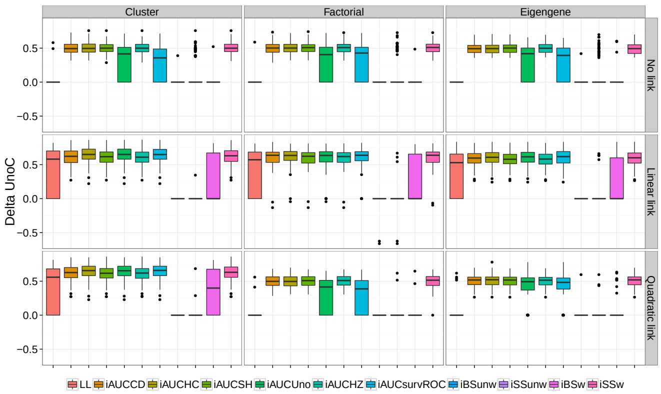

Delta of UnoC (CV criteria − vHCVLL value).
Figure S57 (left): autoPLS−Cox. Figure S58 (right): Cox−PLS.

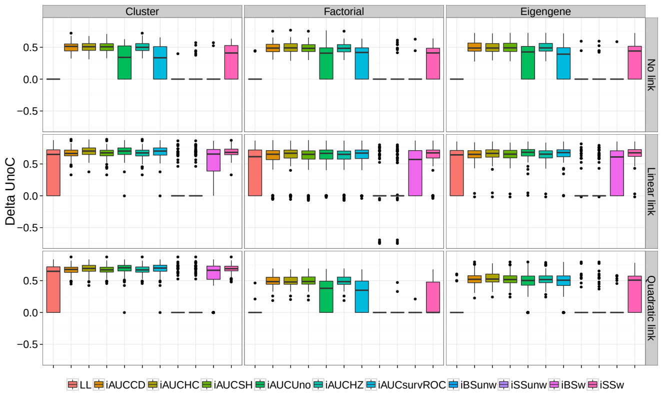

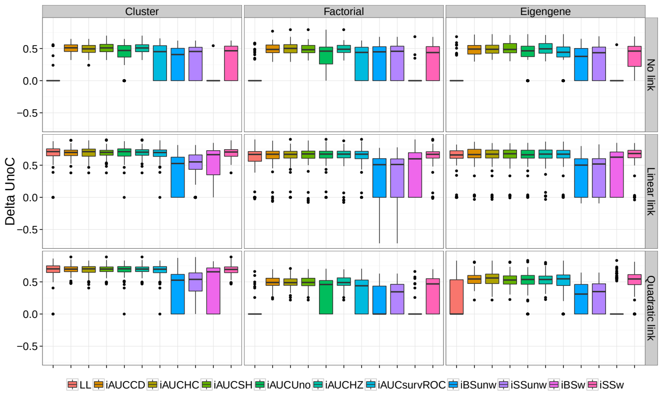

Delta of UnoC (CV criteria − vHCVLL value).
Figure S59 (left): PLSDR. Figure S60 (right): DKPLSDR.

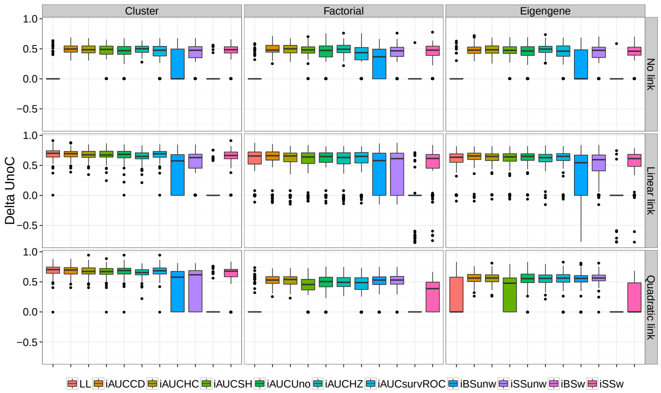

Figure S61: Delta of UnoC (CV criteria − vHCVLL value). DKsPLSDR.

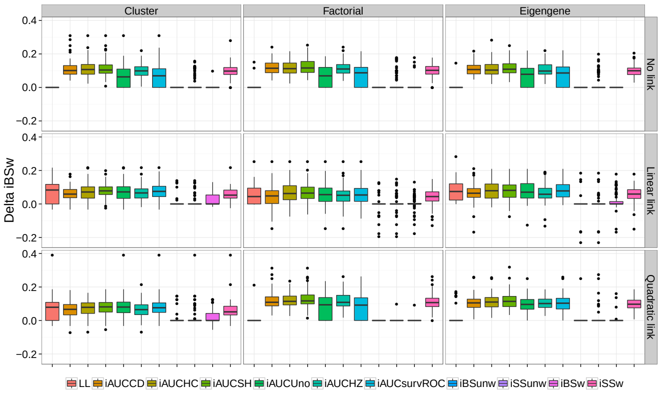

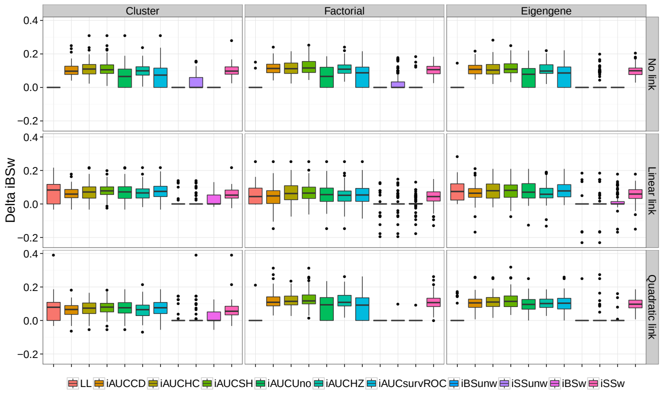

Delta of iBSW (CV criteria − vHCVLL value).
Figure S62 (left): PLS−Cox. Figure S63 (right): autoPLS−Cox.

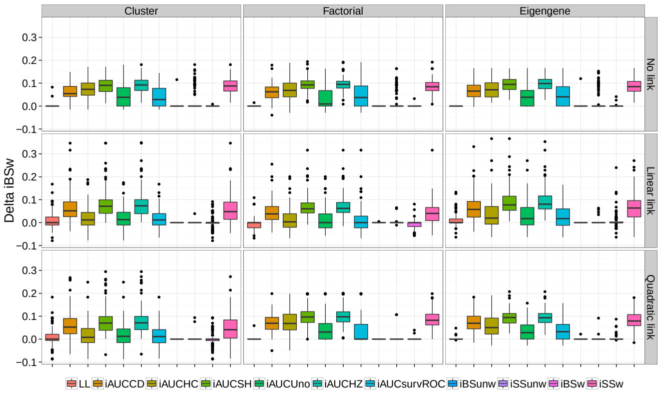

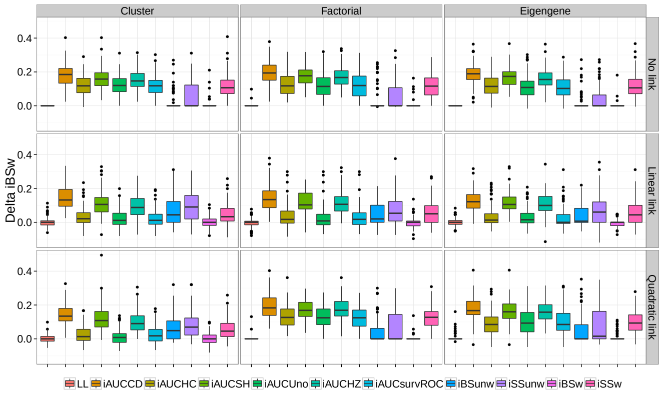

Delta of iBSW (CV criteria − vHCVLL value).
Figure S64 (left): Cox−PLS. Figure S65 (right): sPLSDR.

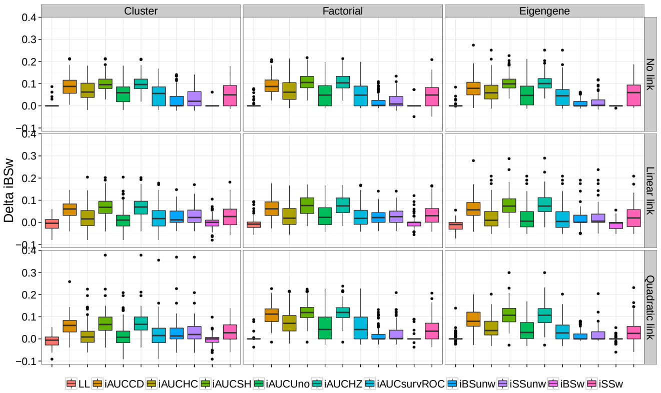

Figure S66: Delta of iBSW (CV criteria − vHCVLL value). DKsPLSDR.

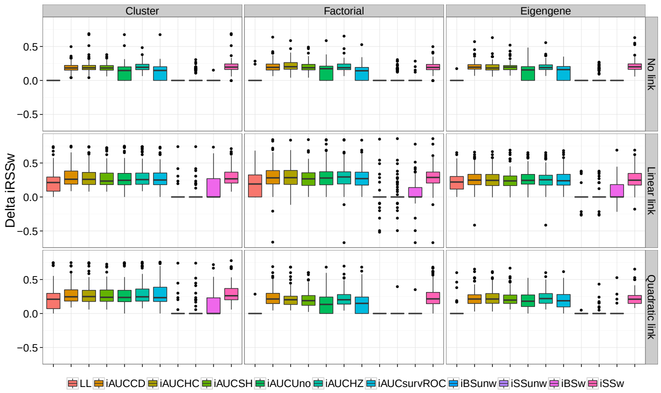

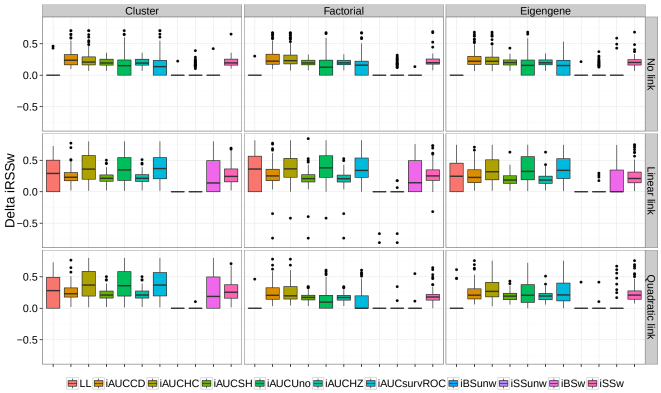

Delta of iRSSW (CV criteria − vHCVLL value).
Figure S67 (left): PLS−Cox. Figure S68 (right): Cox−PLS.

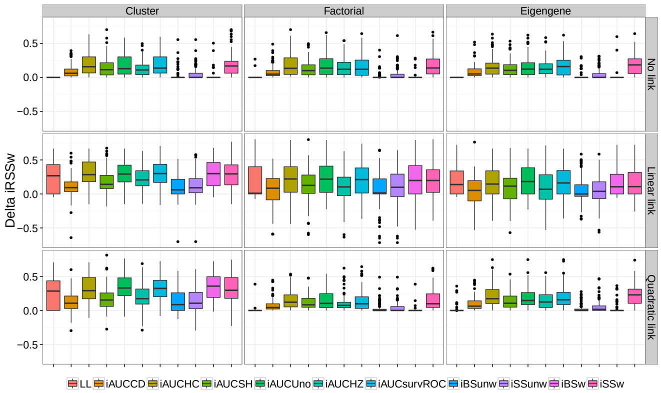

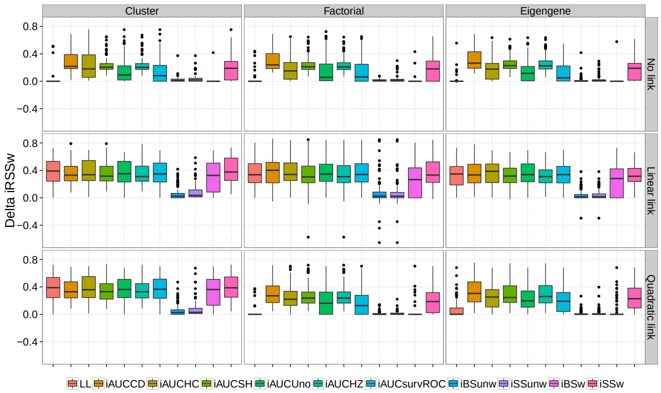

Delta of iRSSW (CV criteria − vHCVLL value).
Figure S69 (left): sPLSDR. Figure S70 (right): DKPLSDR.

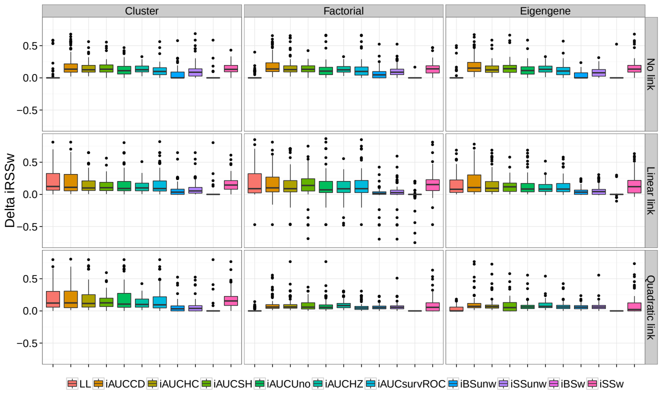

Figure S71: Delta of iRSSW (CV criteria − vHCVLL value). DKsPLSDR.

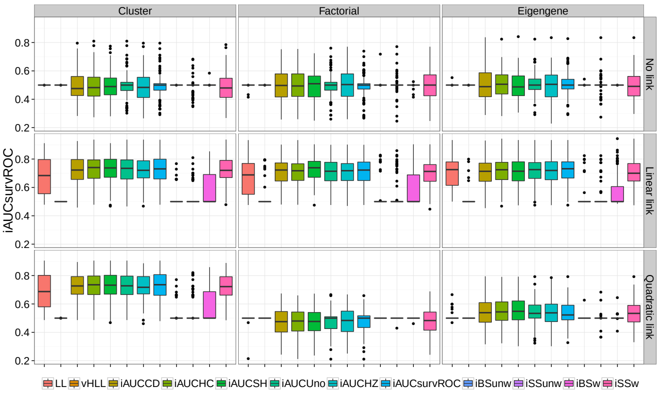

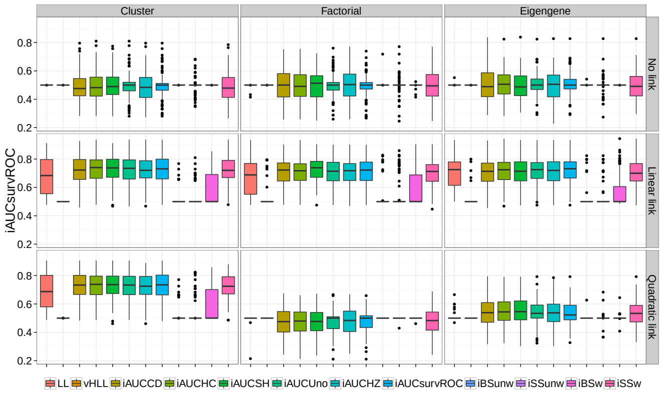

Delta of SurvROC (CV criteria − vHCVLL value).
Figure S72 (left): PLS−Cox. Figure S73 (right): autoPLS−Cox.

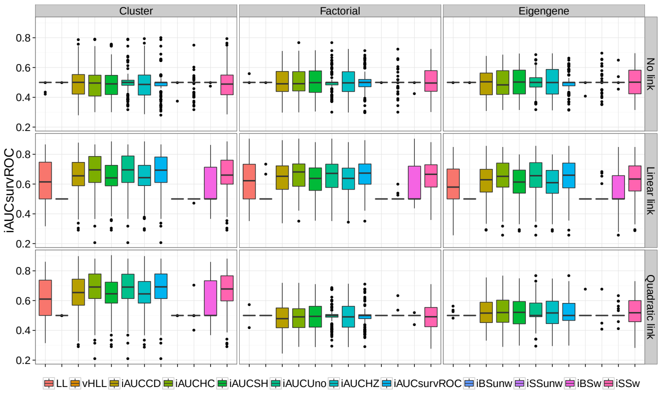

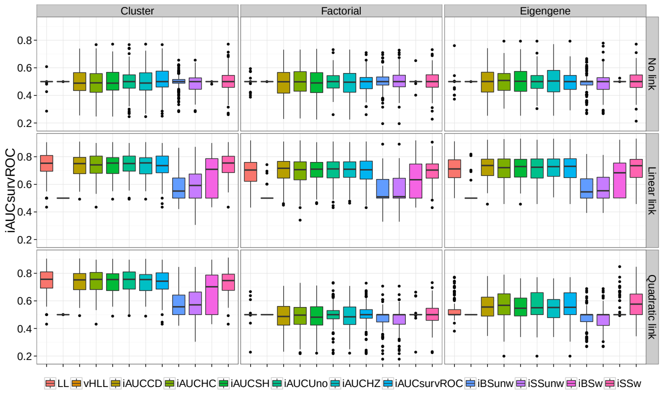

Delta of SurvROC (CV criteria − vHCVLL value).
Figure S74 (left): CoxPLS. Figure S75 (right): DKPLSDR.

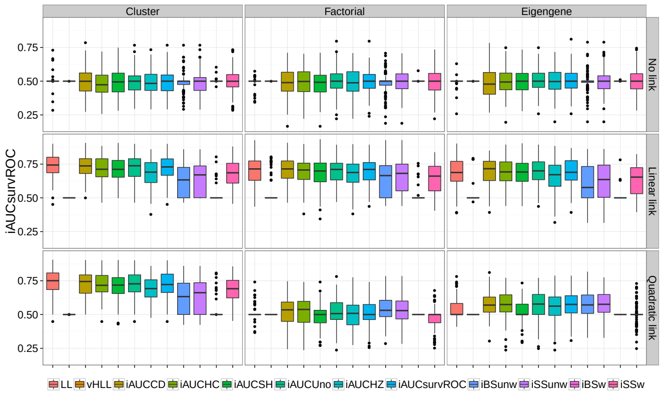

Figure S76: Delta of SurvROC (CV criteria − vHCVLL value), DKsPLSDR.

REFERENCES

Bertrand, F. and Maumy-Bertrand, M. (2021). *Partial Least Squares Regression for Generalized Linear Models*. R package version 1.3.0

Bertrand, F., Meyer, N., and Maumy-Bertrand, M. (2014). *Partial least squares Regression for generalized linear models*. R package version 1.7.0

de Micheaux, P. L., Liquet, B., and Sutton, M. (2019). PLS for Big Data: A unified parallel algorithm for regularised group PLS. *Statistics Surveys* 13, 119 – 149. doi:10.1214/19-SS125

Dejean, S., Gonzalez, I., and Le Cao, K.-A. (2013). *mixOmics: Omics Data Integration Project*. R package version 4.1-4

Liquet, B., de Micheaux, P. L., Hejblum, B. P., and Thiébaut, R. (2015). Group and sparse group partial least square approaches applied in genomics context. *Bioinformatics* 32, 35–42. doi:10.1093/bioinformatics/btv535

Mevik, B.-H., Wehrens, R., and Liland, K. (2011). *pls: Partial Least Squares and Principal Component regression*. R package version 2.3-0

Tenenhaus, M. (1998). *La régression PLS: théorie et pratique* (Paris: Éditions Technip)

Therneau, T. (2013). *A Package for Survival Analysis in S*. R package version 2.37-4

Therneau, T. and Grambsch, P. (2000). *Modeling Survival Data: Extending the Cox Model*. Statistics for Biology and Health (New York: Springer)
